# Microbial succession and dynamics in meromictic Mono Lake, California

**DOI:** 10.1111/gbi.12437

**Published:** 2021-02-25

**Authors:** Alexandra A. Phillips, Daan R. Speth, Laurence G. Miller, Xingchen T. Wang, Fenfang Wu, Patricia M. Medeiros, Danielle R. Monteverde, Magdalena R. Osburn, William M. Berelson, Hannah L. Betts, Reto S. Wijker, Sean W. Mullin, Hope A. Johnson, Victoria J. Orphan, Woodward W. Fischer, Geobiology Course 2017, Geobiology Course 2018, Alex L. Sessions

**Affiliations:** ^1^ Division of Geological and Planetary Sciences California Institute of Technology Pasadena CA USA; ^2^ United States Geological Survey, Earth Systems Process Division Menlo Park CA USA; ^3^ Department of Earth and Environmental Sciences Boston College Chestnut Hill MA USA; ^4^ Department of Marine Sciences University of Georgia Athens GA USA; ^5^ Department of Earth and Planetary Sciences Northwestern University Evanston IL USA; ^6^ Department of Earth Sciences University of Southern California Los Angeles CA USA; ^7^ Department of Biology Harvey Mudd College Claremont CA USA; ^8^ Department of Biological Science California State University Fullerton Fullerton CA USA

**Keywords:** environmental microbiology, geochemistry, limnology, microbial ecology, microbial succession

## Abstract

Mono Lake is a closed‐basin, hypersaline, alkaline lake located in Eastern Sierra Nevada, California, that is dominated by microbial life. This unique ecosystem offers a natural laboratory for probing microbial community responses to environmental change. In 2017, a heavy snowpack and subsequent runoff led Mono Lake to transition from annually mixed (monomictic) to indefinitely stratified (meromictic). We followed microbial succession during this limnological shift, establishing a two‐year (2017–2018) water‐column time series of geochemical and microbiological data. Following meromictic conditions, anoxia persisted below the chemocline and reduced compounds such as sulfide and ammonium increased in concentration from near 0 to ~400 and ~150 µM, respectively, throughout 2018. We observed significant microbial succession, with trends varying by water depth. In the epilimnion (above the chemocline), aerobic heterotrophs were displaced by phototrophic genera when a large bloom of cyanobacteria appeared in fall 2018. Bacteria in the hypolimnion (below the chemocline) had a delayed, but systematic, response reflecting colonization by sediment “seed bank” communities. Phototrophic sulfide‐oxidizing bacteria appeared first in summer 2017, followed by microbes associated with anaerobic fermentation in spring 2018, and eventually sulfate‐reducing taxa by fall 2018. This slow shift indicated that multi‐year meromixis was required to establish a sulfate‐reducing community in Mono Lake, although sulfide oxidizers thrive throughout mixing regimes. The abundant green alga *Picocystis* remained the dominant primary producer during the meromixis event, abundant throughout the water column including in the hypolimnion despite the absence of light and prevalence of sulfide. Our study adds to the growing literature describing microbial resistance and resilience during lake mixing events related to climatic events and environmental change.

**Summary statement**

We investigated microbial succession in hypersaline, alkaline Mono Lake, California, following a climatic perturbation. By combining geochemical and microbiological data, we were able to link depth‐dependent microbial community shifts to limnological changes.

## 1 INTRODUCTION

The interplay between microbial communities, biogeochemical cycling, and changing environmental conditions is of long‐standing interest. Related questions such as the pace and dynamics of microbial succession have been less explored, presumably due to the inherent difficulty of observing natural ecosystems in the midst of dramatic changes. Existing studies of aquatic microbial succession in lakes have focused mainly on loss of stratification, presumably for practical reasons of timing and access. These studies (Hollibaugh et al., 2001; Jones et al., 2008; Shade et al., 2011, 2012) suggested that aquatic communities were generally quite stable in the face of disturbance due to mixing, with high resistance and resilience. We are not aware of any previously published time series examining succession across the onset of stratification and anoxia. Given that climate models and field observations alike suggest future increases in lake stratification with global warming (Arvola et al., 2010; Peeters et al., 2002), this represents a significant knowledge gap. Here, we report a two‐year time series (2017–2018) of microbial diversity and geochemistry from Mono Lake, California, that captures one such transition—the lake's shift from annually mixed (monomictic) to indefinitely stratified (meromictic), following an exceptionally large snowmelt in 2017. This dataset provided a unique opportunity to study both shifts in microbial composition and lake geochemistry that accompany the onset of stratification, as well as the timing of these changes.

**FIGURE 1:**
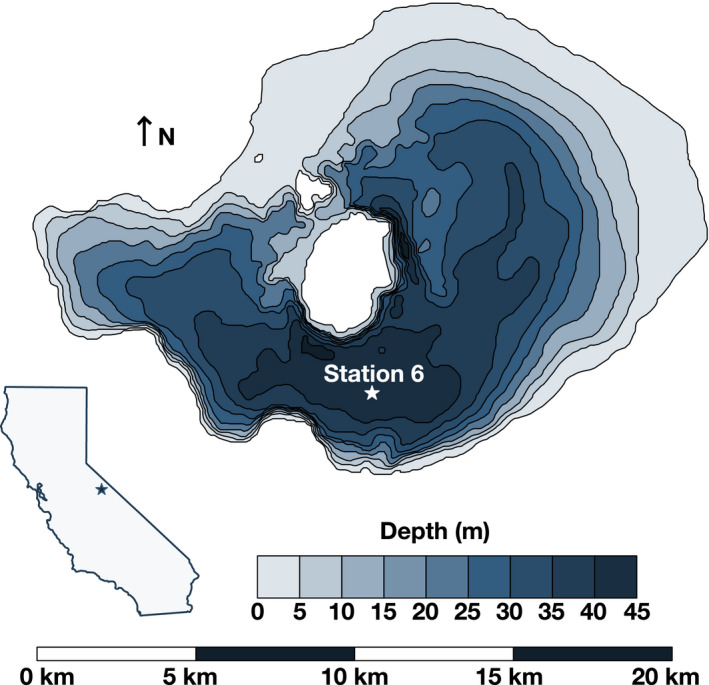
Bathymetric map of Mono Lake after Bruce, Jellison, Imberger, & Melack (2008). Location is indicated on the California map insert with a blue star. All samples were collected from Station 6 in the south basin, marked with a white star. White areas on the map correspond to Paoha island (larger island) and Negit Island to the north

Mono Lake is a hypersaline (~85 ppt, ~3x seawater salinity), alkaline (pH 9.8), closed‐basin lake located in the eastern Sierra Nevada Mountains of California, USA (38°N, 119°W; Figure 1). Water enters the lake primarily from snowmelt via streams (Wilson Creek, Mill Creek, Rush Creek, Lee Vining Creek) and exits by evaporation (~1 m/yr). This hydrologic condition, combined with weathering of Sierran granite in the lake's watershed, produces the high salinity and pH characteristic of Mono Lake (Garrels & MacKenzie, 1967). These properties also tightly tie lake levels and water chemistry to climate, with modern and Pleistocene high stands correlated with wet Sierra Nevada conditions (Benson et al., 1998). Further, primary productivity in Mono Lake is extremely high (269–1060 g C m^−2^ yr^−1^) due largely to the activity of the green alga *Picocystis* sp. strain ML (Jellison & Melack, 1993). The brine shrimp *Artemia monica* is the predominant metazoan predator in Mono Lake, feeding on *Picocystis* and in turn supplying a food source to hundreds of thousands of migrating birds that flock to the lake annually on the Pacific Flyway (Wiens et al., 1993). Thus, despite its modest size and extreme chemistry, Mono Lake serves an essential ecological role beyond the Sierra Nevada. Seasonal surveys of primary production, *Artemia*, and birds such as grebes, gulls, and phalaropes have been conducted since at least 1980 (Cooper et al., 1984; Melack et al., 2017), though they have not provided much insight into Mono Lake's microbial community.

Mono Lake is typically monomictic, with thermally driven summer stratification disrupted by winter, wind‐driven overturn. However, in years with very high spring–summer freshwater inputs, often correlated with El Niño–Southern Oscillation (ENSO) events, a buoyant layer with >10 ppt lower salinity can form at the surface of the lake that withstands winter mixing. This leads to protracted stratification that can continue for five or more years, depending on continued water balance and storm severity. During this time, the hypolimnion becomes euxinic, with sulfide concentrations reaching hundreds of micromolar (Miller et al., 1993). For example, following the deep snow pack associated with the 1982 ENSO, summer melting brought a 2.6 m lake level rise that triggered persistent stratification from 1983 through 1989 (Melack et al., 2017). Mono Lake was again meromictic from 1996 to 2004 and from 2006 to 2008. It has since remained monomictic until the exceptional snow year of 2017 when our study began.

Although there have been numerous studies of Mono Lake, none have yet captured the microbial response to the onset of meromixis with high‐throughput sequencing. Hollibaugh et al. (2001) quantified bacterial clades using denaturing gradient gel electrophoresis (DGGE) but found no evidence for microbial community restructuring between 1994 and 1995 when the lake became meromictic. Further DGGE studies conducted between 2000 and 2006, as part of the Mono Lake Microbial Observatory, cataloged responses of particular metabolic clades including aerobic methanotrophs (Carini et al., 2005), sulfate reducers (Scholten et al., 2005), nitrifiers (Carini & Joye, 2008), and methylotrophs (Nercessian et al., 2005). Recently, two studies of Mono Lake have used next‐generation sequencing technology, including 16S rRNA gene amplicon sequencing, environmental metagenomics, and transcriptomics (Edwardson & Hollibaugh, 2017; Stamps et al., 2018), but these investigations focused on single time points. To supplement this literature, we monitored Mono Lake at the onset of a new meromictic period that began in 2017. Our two‐year (thus far) time series reflects six seasonal sampling dates with physical, chemical, and biological data to characterize Mono Lake during this transition.

## 2 METHODS

### 2.1 Water sampling

**FIGURE 2:**
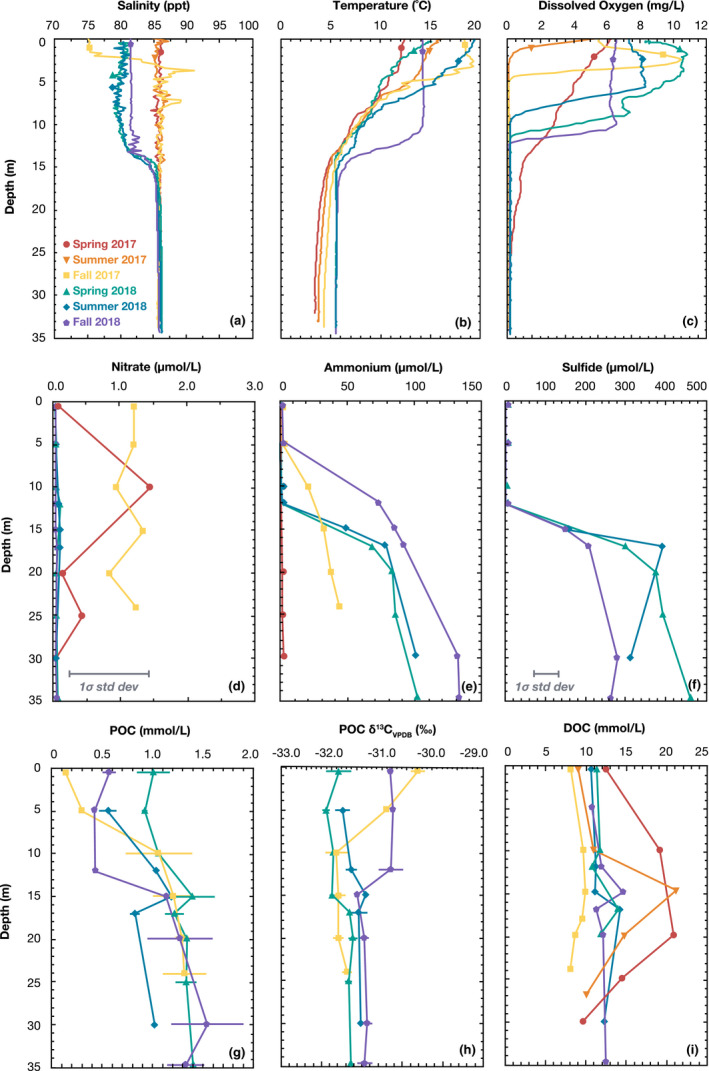
Vertical profiles of (a) salinity, (b) temperature, (c) dissolved oxygen, (d) nitrate, (e) ammonium, (f) sulfide, (g) particulate organic carbon (POC) (h), POC δ^13^C_VPDB_, and (i) dissolved organic carbon (DOC) concentrations at Station 6, Mono Lake, CA. Profiles were measured in spring 2017 (red circles), summer 2017 (orange inverted triangles), fall 2017 (yellow squares), spring 2018 (green triangles), summer 2018 (blue diamonds), and fall 2018 (purple pentagons)

All water sampling was conducted at “Station 6” (37.95739 N, −119.0316 W), a representative site in the deepest part of the south basin, marked by a buoy (Figure 1). Samples were collected on May 23, June 22, and September 19, 2017 (hereafter referred to as spring 2017, summer 2017, and fall 2017, respectively), and May 8, June 13, and October 9, 2018 (spring 2018, summer 2018, and fall 2018, respectively). Sampling was preceded by measurements of conductivity, temperature, dissolved oxygen, and photosynthetically active radiation (PAR) using a SeaBird SBE 19 CTD cast to 35 m depth. Salinity profiles were initially calculated from conductivity after Jellison et al. (1991). While this produced accurate bottom‐water salinity estimates (as compared to handheld refractometer measurements of 86 ± 2 ppt; Atago S‐28E), it also yielded sharp fluctuations in salinity across the thermocline, which we do not believe are physically plausible and which made interpreting the structure of the chemocline difficult. Instead, we used the default SeaBird seawater calibration of our CTD, then scaled each profile linearly to a bottom‐water salinity of 86. This produced a smoother chemocline structure (Figure 2a), but potentially at the expense of less accurate surface salinity values. Discrete water samples were collected using a 5‐L Niskin bottle or a 2” diameter submersible well pump at five to eight depths. When the pump was used, water flowed through the pump and tubing at each depth for at least two minutes before sampling. *Artemia monica* (brine shrimp) were removed from sampled water using a 10‐µm Nitex‐screen filter. Water aliquots were taken for dissolved inorganic carbon (DIC), dissolved organic carbon (DOC), dissolved organic nitrogen (DON), sulfate and sulfide, nutrients and major ions, particulate organic carbon (POC) and fatty acids (FAs), 16S rRNA gene amplicon sequencing, and incubations. Not all parameters were sampled on all dates, as described in Table S1.

### 2.2 Geochemical measurements

#### 2.2.1 Sulfide and sulfate

Water (7 ml) was immediately preserved for sulfide analysis by filtering through a 0.22‐µm polyvinylidene difluoride (PVDF) syringe filter into a 15‐ml falcon tube containing 3 ml zinc acetate (1 M), precipitating zinc sulfide and zinc carbonate. Samples were kept at room temperature until analysis. Concentrations were measured using a modified spectrophotometric assay (Cline, 1969) at Caltech, described in detail in the SI. Replicate measurements yielded typical 1σ standard deviations of 30 µM.

Water (10 ml) was subsampled on the boat for dissolved sulfate by filtering through a 0.22 µm PVDF syringe filter into a 15‐ml falcon tube containing 200 µl hydrochloric acid (3 M). HCl was added to volatilize H_2_S and to suppress biological activity. Samples were stored at room temperature until analysis. Sulfate concentrations were measured with a Dionex Ion Chromatography System 2000 equipped with an AS29‐Fast (4 µm diameter) column at Caltech, yielding 1σ standard deviations of 1.5 mM for sample replicates (~1.2% rsd).

#### 2.2.2 Nutrients and major ions

Water (50 ml) from the POM filtrate (see below) was subsampled on shore into 50‐ml falcon tubes and frozen at −20°C until shipment on dry ice to Scripps Institution of Oceanography Nimitz Marine Facility for nutrient analysis. There, phosphate, silica, nitrate, nitrite, and ammonium concentrations were analyzed by modified colorimetric assays (Armstrong et al., 1967; Bernhardt & Wilhelms, 1967) on a SEAL Analytical segmented continuous‐flow AutoAnalyzer 3. Nutrient standards yielded 1σ standard deviations of 1 µM for nitrate, phosphate, and ammonium and 0.01 µM for nitrite and silica. For cation measurements, an additional 15 ml of filtered water was subsampled into 15‐ml falcon tubes and kept frozen until analysis. Concentrations of K^+^, Mg^2+^, Ca^2+^, Si^4+^, Sr^2+^, Li^2+^, Fe^2+^, and Mn^2+^ were analyzed on a Thermo Element2 high‐resolution inductively coupled plasma mass spectrometer (HR‐ICP‐MS) at the University of Southern California, using external calibration. The calibration standards had major ion ratios similar to Mono Lake water. IAPSO (International Association for the Physical Sciences of the Ocean) standard seawater was analyzed as a reference.

#### 2.2.3 Dissolved inorganic carbon (DIC)

Water for DIC analysis was collected directly from the Niskin bottle on the boat and filtered through a 0.22‐µM polyether sulfone (PES) syringe filter into 15‐ml falcon tubes until overflowing. For analysis, 0.1–0.4 g water was injected into an evacuated, 10‐ml Exetainer and 5 ml of 10% phosphoric acid was added. The evolved CO_2_ was analyzed following Subhas et al. (2015). Exetainer gases were purged with N_2_ flushing through a mass control valve into a mixing bag (Liaison‐Picarro). Gas was fed via N_2_ carrier to a Picarro Cavity Ring Down Spectrometer (G2131‐i) at University of Southern California for analysis of total ^12^C and ^13^C. Weighed CaCO_3_ working standards were used for calibration. Replicate samples yielded 1σ standard deviations of ±4 µM DIC (at 2000 µM) and 0.15‰ for δ^13^C_VCDT_.

#### 2.2.4 Dissolved organic carbon (DOC)

On the boat, 500 ml of water was subsampled for DOC analysis into acid‐cleaned 1‐L amber bottles (Nalgene, HDPE) and placed on ice. Directly after returning to the lake shore, this water was filtered through pre‐washed 0.22 µm PES syringe filters into acid‐washed 60‐ml amber bottles (Nalgene) and frozen at −20°C. Immediately before analysis, samples were thawed and acidified to pH 2 with 10 M hydrochloric acid (HCl). DOC concentrations were measured with a Shimadzu TOC‐L_CPH_ analyzer at University of Georgia. Potassium hydrogen phthalate was used as a standard for the DOC calibration curve (prepared the same day). Internal blanks and ultrapure water (>18.2 MΩ) blanks were also analyzed at the same time. Analytical accuracy and precision were tested against the Consensus Reference Material after Hansell (2005). 1σ standard deviations of triplicate samples varied but were never above 0.15 µM.

#### 2.2.5 Dissolved organic nitrogen (DON)

Water samples for total DON were collected and filtered through 0.22 µM PES syringe filters into 15‐ml falcon tubes and placed on ice on the boat. Total dissolved nitrogen (TDN) concentrations were measured by oxidizing all reduced N species to nitrate with alkaline persulfate solution (Nydahl, 1978; Wang et al., 2015), followed by quantification of the resulting nitrate with the chemiluminescence method (Braman & Hendrix, 1989) at Princeton. In a pre‐combusted 4‐ml glass vial, 0.1 ml of Mono Lake water was combined with 1 ml persulfate oxidizing reagent (1 g recrystallized low‐N K_2_S_2_O_8_ and ACS grade 1.5 g NaOH in 100 ml ultrapure water) and autoclaved for 1 hr. DON concentration was calculated by subtracting the concentrations of nitrate and ammonium/ammonia from the concentration of TDN. The nitrogen concentration of blanks (persulfate oxidizing reagent) was <0.3 μM. 1σ standard deviations for replicate DON concentration measurements were <2%.

#### 2.2.6 Particulate organic matter (POM)

Water (3–5 L) from each depth was collected in acid‐cleaned, 10‐L high‐density polyethylene (HDPE) carboys on the boat. Within three hours after sampling, ~2 L subsamples from these jugs were filtered on shore using a Cole‐Parmer Masterflex I/P peristaltic pump (model 77602‐10) through pre‐combusted 0.7 µm × 147 mm OD GF/F filters in a Whatman stainless‐steel filter holder via Masterflex Tygon E‐LFL pump tubing (I/P 73, 9.5 mm ID). Filters containing POM were rinsed with ~1 L of 85 g/L NaCl salt solution in ultrapure water to remove dissolved carbonate and sulfate and then frozen until lyophilization. Freeze‐dried filters were subsampled for POC via 1/16 inch diameter hole punch. Filter punches were exposed to fumes of 12 M HCl for one week to remove DIC. Three decarbonated hole punches were folded into 9 mm × 5 mm tin capsules (OEA Labs) and combusted to CO_2_ with a Thermo Fisher Scientific Flash Elemental Analyzer (EA) Isolink CN connected to a Delta V Plus Isotope Ratio Mass Spectrometer (IRMS) via a ConFlo IV Universal Interface at Caltech. A calibrated in‐house standard (urea, δ^13^C_VPDB_ = −27.8‰) was used for isotope calibration and concentration calculations. 1σ standard deviations were variable but typically ~0.10 mM, and are plotted as the error bars of Figure 2g.

#### 2.2.7 Fatty acids (FAs)

Cultured *Picocystis* biomass and Mono Lake POM were analyzed for fatty acid compositions via gas chromatography–mass spectrometry (GC‐MS) at Caltech. *Picocystis* biomass (~10 mg) was freeze‐dried, then ground in a solvent‐washed mortar and pestle. Freeze‐dried POM filters were cut in half, with one half archived while the other was extracted. Samples were extracted and derivatized as methyl esters following standard protocols described in the SI.

### 2.3 Microbiology

#### 2.3.1 *Picocystis* cultures

We obtained *Picocystis* sp. strain ML pure culture (Roesler et al., 2002) from R. Oremland (USGS) and grew cells in batch culture under white light on a rotary shaker at 180 rpm and 30°C. L1 minimal medium was prepared and used as described in Guillard and Hargraves (1993), substituting artificial seawater (35 g/L Instant Ocean Sea Salt) in place of filtered natural seawater. The growth medium was supplemented with salt to reach 70 g/L NaCl. Cell growth was monitored by measuring absorbance at 600 nm, and purity was checked by microscopy. Cultures were harvested in the late exponential phase (OD 0.4–0.6) and stored at −25°C until lyophilization, extraction, and analysis.

#### 2.3.2 Sulfate reduction incubations

Incubations for sulfate‐reducing potential were started in fall 2018 by addition of 30 ml Mono Lake water from 12, 17, 20, and 35 m water depth to N_2_‐sparged 60‐ml serum bottles with blue butyl stoppers at the time of sampling. Samples were kept on ice overnight until return to Caltech, when they were incubated at 4°C in the dark. Incubations were conducted in duplicate with four conditions: lactate addition (2 mM final concentration), pyruvate addition (2 mM final concentration), no addition, and killed control. Killed controls were autoclaved 48 hr after sample addition. Incubations were monitored by measuring dissolved sulfide concentration using the Cline assay (described in SI) at three days, two weeks, one month, and six months.

#### 2.3.3 16S rRNA Illumina TAG sequencing

Water samples for high‐throughput Illumina 16S rRNA gene amplicon sequencing were collected in 50‐ml falcon tubes and stored on ice or at 4°C for ~24 hr until benchtop centrifugation (5250 *g*, 20 min, 4°C). Supernatant was discarded until a pellet and ~1 ml liquid remained. Pellets were resuspended and transferred to 2‐ml tubes and centrifuged (16 000 *g*, 10 min). Supernatant was decanted, and pellets were kept at −20°C until DNA extraction following Zhou et al. (1996; details available in the SI). Following the initial PCR, amplicons were barcoded using Illumina Nextera P5/P7 primers and pooled prior to sequencing and subsequent sample demultiplexing at Laragen Inc. Sequence data were analyzed using QIIME 1.8.0 (Caporaso et al., 2010), with a 99% OTU cutoff and taxonomic assignment using the SILVA138 NR99 database (Quast et al., 2013). OTU counts were binned by taxonomic assignment at the genus rank. Using R, counts for each genus were normalized to relative abundance per dataset. After normalization, chloroplasts (representing mainly the eukaryote *Picocystis*) and fully unassigned sequences (<90% identity to any SILVA sequence) were removed for clarity, and the data were binned in 3 groups: genera represented by >1% of the sequences of any one sample, genera represented by 0.1%–1% of the sequences of any one sample, and genera not present in more than 0.1% in any sample. The resulting abundance tables were used to generate heat maps in MATLAB (code in SI). Statistical analyses (NMDS, ANOSIM, ADONIS, Pearson correlation, SIMPER) were carried out in R using the *vegan* package (Oksanen et al., 2019). Alpha diversity was analyzed by rarefying each sample to 1,370 sequence depth using QIIME 1.8.0 and calculating richness (number of distinct OTUs), evenness (Shannon equitability), and diversity (Shannon–Wiener Index) following Shannon and Weaver (1949).

## 3 RESULTS

### 3.1 Physical and chemical properties

Salinity was vertically well mixed (~86 ppt) in spring and summer 2017 (Figure 2a). A lens (~1 m) of less saline (75 ppt) surface water developed by the fall. Mono Lake typically overturns in the winter, but the lake remained stratified throughout 2018. Temperature structure exhibited seasonable variability, although the base of the thermocline remained at ~15 m (Figure 2b). Dissolved oxygen profiles (Figure 2c) indicated that in spring 2017 at the start of our time series, the lake was oxic at the surface and dysoxic at depth. In summer 2017, a significant oxycline developed at 3 m, which progressively deepened throughout our time series. After and including summer 2017, no detectable O_2_ was measured below the chemocline. These profiles demonstrate monomictic conditions in the spring and summer of 2017 with a shift to meromixis in the winter of 2017–18.

Dissolved sulfate (~105 mM), silica (540 ± 60 µM), and phosphate (697 ± 44 µM) remained high throughout the time series (Table S2), while nitrite remained very low (<0.3 µM). In contrast, nitrate, ammonium, and sulfide concentrations changed with prolonged stratification. Nitrate decreased from its maximum of 1.45 µM in fall 2017 to <0.01 µM by fall 2018 (Figure 2d). Ammonium (Figure 2e) was low (<4 µM) in spring 2017, while the lake was fully mixed, but gradually increased below the chemocline, reaching 141 µM by fall 2018. Sulfide was not measured in 2017, but throughout 2018, it accumulated below the chemocline, reaching a maximum concentration of 464 µM (Figure 2f). Major cations (K^+^, Mg^2+^, Ca^2+^, Si^4+^, Sr^2+^, Li^2+^, Fe^2+^, Mn^2+^), DIC concentrations, and DIC δ^13^C_VPDB_ values were measured in spring 2017 and are described in the SI; these species are sufficiently concentrated that they are presumed not to change much over timescales of a few years.

POC was high (~1 mM) and increased with depth (Figure 2g). No previous studies have reported POC for comparison, but these concentrations align with Mono Lake's high primary productivity. The δ^13^C_VPDB_ values of POC in the hypolimnion were roughly constant at −31.6 ± 0.3‰ (Figure 2h), whereas in the epilimnion, they were variable, with values of −30.2 ± 0.1‰ in fall 2017 and −30.8 ± 0.1‰ in fall 2018. Dissolved organic carbon (DOC) was also very high and ranged from 8.13 to 21.31 mM (Figure 2i). Notably, although inorganic fixed nitrogen was very low (Figure 2d, Table S2), DON was elevated (~0.3 mM; Table S2).

**FIGURE 3:**
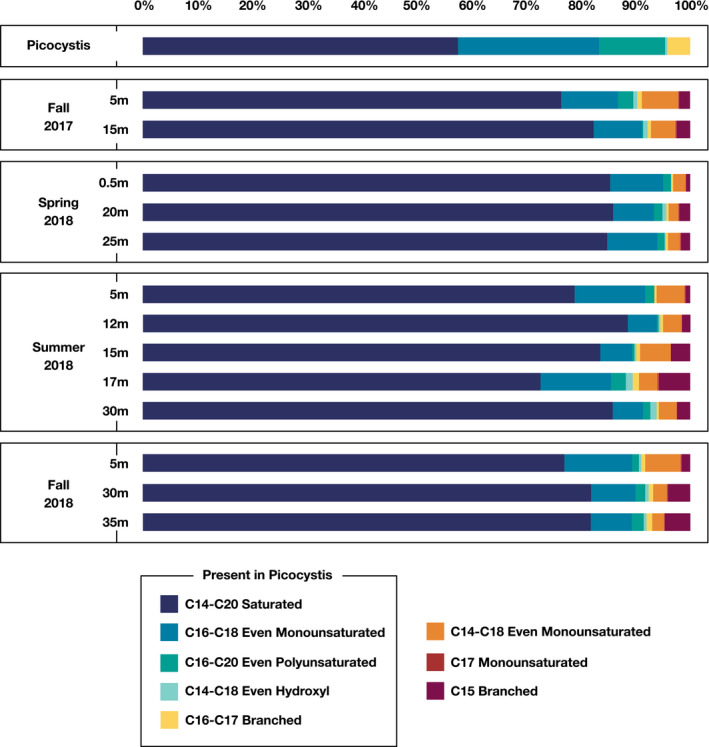
Relative abundance of fatty acids (FAs) detected in *Picocystis* cultures and Mono Lake water‐column POC, analyzed as fatty acid methyl esters. For the purpose of comparison, FAs are grouped into five categories shared between *Picocystis* and POC extracts: C_14_–C_20_ saturated (dark blue), C_16_ and C_18_ monounsaturated (turquoise), C_16_, C_18_, and C_20_ polyunsaturated (green), C_14_, C_16_, and C_18_ β‐hydroxy (light green), and C_16_ and C_17_ branched (yellow). Categories of FAs unique to POC samples included C_14_, C_16_, and C_18_ monounsaturated (orange), C_17_ monounsaturated (red), and C_15_ branched (maroon)

The total hydrolyzable fatty acid (FA) content of cultured *Picocystis* sp. strain ML is shown in Figure 3 and described in detail in the SI. Extractable fatty acids from Mono Lake POM were consistent with *Picocystis* being the main source (Figure 3, Table S3). Saturated FA between C_14_ and C_20_ accounted for a slightly larger normalized abundance than observed in *Picocystis* culture, potentially reflecting significant contributions from bacterial sources and/or degradation of short‐lived double bonds (Rontani et al., 2012). Monounsaturated FAs between C_14_ and C_16_ not synthesized by *Picocystis* varied between 2% and 4% in the hypolimnion and reached up to 7% in the epilimnion. Iso and anteiso‐C_15_ FAs were uniquely present in POM and reached up to 5%–6% in hypolimnion samples. Long‐chain fatty acids (>C_20_) were also identified in variable quantities (~1–10 ng/ml); these molecules were likely sourced from terrestrial or littoral vegetation and were removed before normalization (Table S3).

### 3.2 Microbial community composition

**FIGURE 4:**
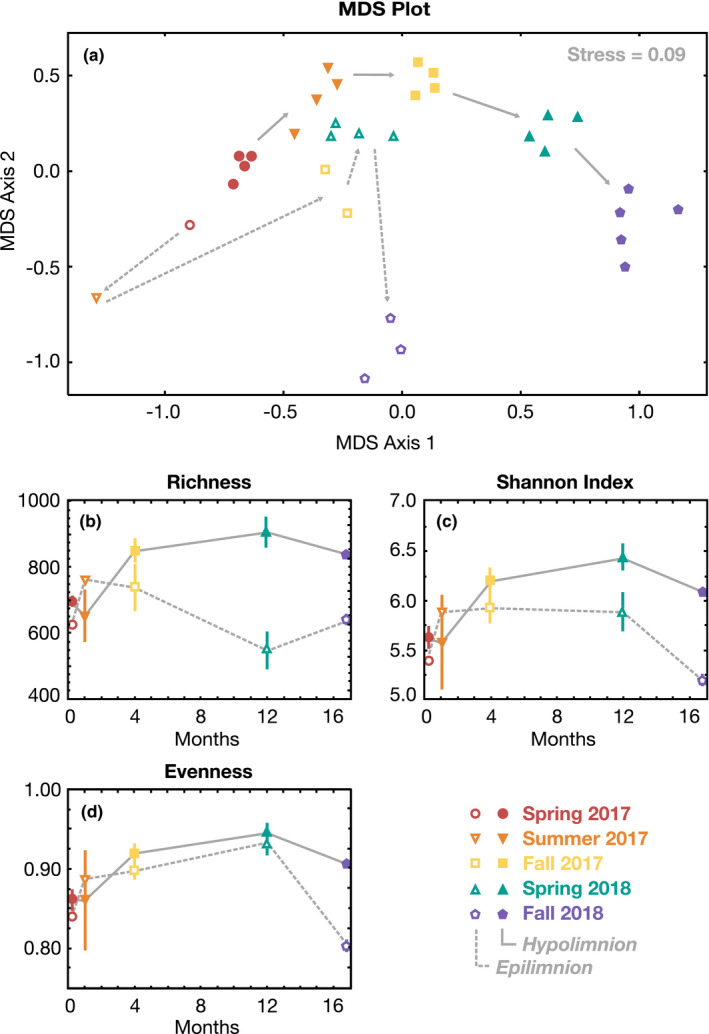
Visualizations of 16S rRNA gene amplicon data. Epilimnion (surface water) and hypolimnion (deep‐water) samples are indicated by open and closed symbols, respectively. Symbols and colors are the same as in Figure 2. Shallow and deep samples from spring 2017 are coded as epilimnion and hypolimnion, respectively, although the thermocline was only weakly developed at that time. Dashed arrows indicate the time trajectory of the epilimnion microbial community while solid arrows indicate the hypolimnion. (a) Multidimensional scaling (MDS) plot of all 32 samples across the two‐year time series, where distance across either axis represents degree of dissimilarity. (b) Richness (alpha diversity). (c) Shannon diversity index. (d) Species evenness. In panels b–d, values are averaged across all epilimnion or hypolimnion samples for each time point with bars representing standard deviations

The similarity in 16S rRNA gene amplicon diversity for our time‐series dataset was visualized using multidimensional scaling (MDS) analyses (Figure 4a). In spring 2017, samples from all depths hosted similar microbial community composition, but with stratification, the communities in the epilimnion and hypolimnion diverged and remained distinct for the rest of the time series. Indeed, when the epilimnion and hypolimnion communities were considered separately, time was the overwhelming driver of variation, with surface water ANOSIM R = 0.9822 (*p* = .001) and deep‐water R = 0.9251 (*p* = .001; ANOSIM R value is a metric describing the ratio between within‐group and between‐group ranked Bray–Curtis community dissimilarities, typically ranging from 0 to 1 with increasing correlation). The Pearson correlation of time and dissimilarity was also high in the epilimnion (0.5801, Mantel test *p*‐value = .002) and hypolimnion (0.8508, *p* = .001). When samples were processed together, time explained less of the variation (R = 0.5316, *p* = .001), implying that different depths in the water column had independent trajectories. Hypolimnion and epilimnion samples also exhibited distinct patterns in species richness, Shannon diversity, and evenness (Figure 4b–d; Table S4), as described in the SI.

**FIGURE 5:**
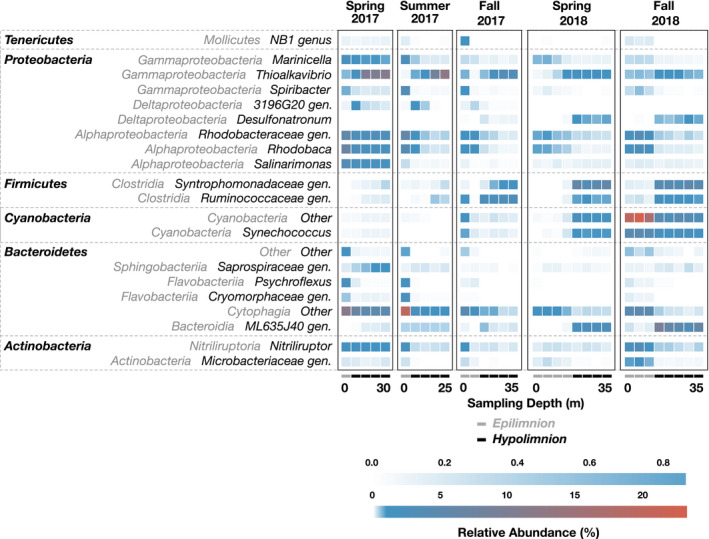
Heatmap of 16S rRNA sequence relative abundance for genera >1.0% at Station 6. Sampling time points are ordered with increasing depth from left to right within each box, and with increasing time from left to right between boxes. Epilimnion and hypolimnion samples are indicated with gray and black rectangles below each column, respectively. Amplicon sequences are binned at the genus level, with phylogenetic classes shown in gray italics to the left of the corresponding genus, and phyla indicated on far left in bold

**FIGURE 6:**
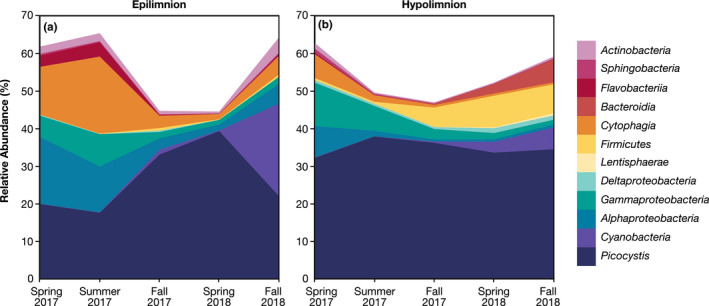
Relative abundances of bacterial 16S rRNA sequences, grouped by class or phylum, in the (a) epilimnion (b) and hypolimnion. *Picocystis* (dark blue) abundance was inferred from chloroplast sequences. Some phyla were not included, as their relative abundance was too low to be discernable in this visualization method. These groups included the following: Euryarchaeota, Actinobacteria, Deinococcus–Thermus, Planctomycetes, Spirochaetes, Verrucomicrobia, and Tenericutes

Almost all observed phenotypes belonged to Bacteria (Figure 5, Figure S2). The uncultured lineage DHVEG6 was the only Archaeal group recovered at >0.1%, found in a single sample from fall 2018. Previous studies reported similar bacterial dominance in moderately hypersaline lakes, with Archaea frequently dominating under extremely high salinity (Jiang et al., 2006; Oren, 2008). 98.7% of chloroplast sequences were assigned to the dominant phototrophic alga, *Picocystis*. We therefore used chloroplast relative abundance as a proxy for *Picocystis*, which varied between 17% and 40% relative abundance in the epilimnion (Figure 6a; Figure S1a) and 32%–38% in the hypolimnion (Figure 6b; Figure S1). *Picocystis* was the only organism consistently abundant throughout the water column at all time points. Abundances of other taxa, described in more detail in the SI, varied strongly between epilimnion and hypolimnion samples (Figures 4, 5, 6, Figure S2), with ANOSIM R value = 0.2515 (*p*‐value = .002). A one‐way ANOSIM with all samples indicated that time was another significant driver of variation in our data (R = 0.5316, *p* = .001); however, seasonality had a smaller effect (R = 0.1553, *p* = .02) implying that ongoing stratification and not necessarily the time of the year influenced the observed changes in microbial community composition.

### 3.3 Sulfate reduction incubations

**FIGURE 7:**
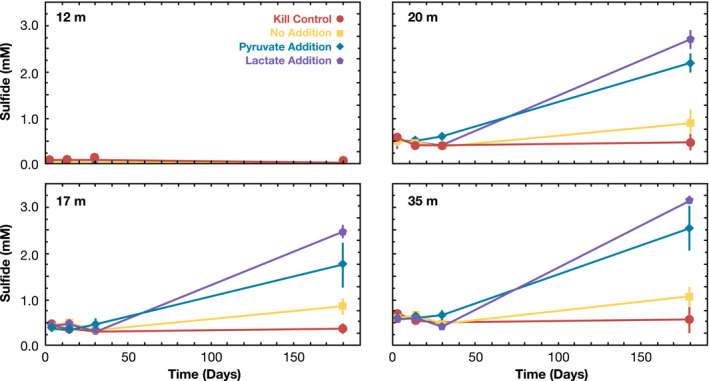
Measurements of water‐column sulfate reduction potential in incubation experiments. Samples were collected in spring 2018 from 12, 17, 20, and 35 m water depths. Each depth was monitored for sulfide production over the course of six months. Values are the average of replicates with vertical error bars displaying 1σ standard deviations. The 12 m sample was from the epilimnion, while other samples were collected from the hypolimnion and at time zero began with background sulfide concentrations in the lake (0.5 mM). 2 mM lactate addition (purple pentagons), 2 mM pyruvate (blue diamonds), no addition (yellow squares), and the autoclaved killed control (red circles)

Sulfide concentrations in the lake water incubations are shown in Figure 7. Incubations with water from 12 m depth (above the chemocline) demonstrated no sulfide production across all conditions and time points. Samples from below the chemocline began with sulfide concentrations at ~0.5 mM. After six weeks, sulfide concentrations were unchanged (~0.5 mM) in all conditions, suggesting that sulfate reduction in situ was very slow at best. After six months, all incubations of samples from below the chemocline, with the exception of killed controls, produced significant sulfide. Additional experiments amended with pyruvate and lactate as the carbon source yielded the highest sulfide concentrations. Extrapolation between the last two time points of the no‐addition culture yields a minimum sulfate reduction rate of 0.12 µmol L^−1^ day^−1^. Given the long lag phase of the incubation, it is unclear how applicable this value is to Mono lake itself, but it nevertheless emphasizes that sulfate reduction rates in the water column are likely very slow.

## 4 DISCUSSION

Our dataset for Mono Lake offers insight into aquatic microbial succession during the onset of meromixis. Within just one month of stratification, community shifts were apparent, and 16 months later, these successional dynamics were still occurring (Figures 4, 5, 6). The epilimnion and hypolimnion microbial communities followed distinct paths (Figures 4 and 6), and—with the possible exceptions of *Picocystis* and Cyanobacteria—there appeared to be relatively little biological exchange of these communities across the chemocline. We therefore considered the following four aspects of our data: (a) the dynamic succession in the epilimnion, (b) the more predictable succession patterns in the hypolimnion, (c) the temporal and limnological drivers of sulfur cycling, and (d) the enigmatic ecophysiology of Mono Lake's prolific primary producer, *Picocystis*, and its resistance to stratification.

### 4.1 The dynamic surface community

Mono Lake's epilimnion bacterial community experienced drastic changes throughout our two‐year time series, demonstrating low resistance and resilience (Figures 4a and 6a). Other surveys of stratified lakes have also found that epilimnion bacteria were more variable over time than hypolimnion populations (Shade et al., 2008), reasoning that surface communities face more variability from climatic interactions. Indeed, seasonal Mono Lake surface water temperatures fluctuated between ~0°C and 20°C and winter storms increased terrestrial organic matter delivery, as inferred from increased long‐chain fatty acids (>C_20_) (Table S3). Taken together, it was perhaps unsurprising that Mono Lake's surface microbial community shifted as lake stratification stabilized. What was unexpected is the direction of this shift: a reduction in relative numbers of heterotrophs, with concomitant increase in primary producers.

At the start of our time series, during monomixis, the epilimnion microbial community was characterized by the phototrophic alga *Picocystis* (~20% relative abundance) and aerobic or aerotolerant heterotrophic bacteria (~40% relative abundance) within Alphaproteobacteria, Gammaproteobacteria, Cytophagia, Actinobacteria, Bacteroidia, and Flavobacteriia (Figures 5 and 6a, Figure S2; see SI for taxa specific details). This early epilimnion diversity comprised a suite of saline‐ and alkaline‐adapted heterotrophic bacteria likely capable of degrading and remineralizing complex organic matter such as that supplied by the dominant *Picocystis*.

The drastic shoaling of the oxycline in spring–summer 2017 (Figure 2c) reduced the niche for aerobic metabolisms, but the composition of the epilimnion microbial community was only minimally altered (Figures 4, 5, 6). By fall 2017, however, putative heterotrophs had fallen drastically to ~10% relative abundance and would remain at that level throughout the remainder of our time series. Simultaneously, from fall 2017 through spring 2018, there was a 15%–20% relative increase in Picocystis. By fall 2018, a bloom of phototrophic, non‐diazotrophic, Cyanobacteria appeared, dominated by a genus related to Group 1 species within the marine *Prochlorococcus* and *Synechococcus* clade. This OTU reached ~30% relative abundance, causing epilimnion evenness to drop from 0.93 to 0.80 from spring to fall 2018 (Figure 4d). C_14_–C_18_ monounsaturated fatty acids also increased in the epilimnion during this phototrophic bloom (orange bars, Figure 3) and were tentatively attributed to the Cyanobacteria. Group 1 cyanobacterial species, like those in the genus *Synechococcus*, are up to 50% C16:1 fatty acid by weight (Murata et al., 1992). As this cyanobacterial bloom is represented in just one time point, we do not know whether it will be a persistent feature of the meromictic lake. Regardless, we observed a significant shift in the epilimnion microbial population from one dominated by heterotrophs during monomixis to one dominated by primary producers during meromixis.

What mechanisms allowed Cyanobacteria to thrive in fall 2018 when these OTUs were minimally present throughout the rest of the time series? Classically, cyanobacterial blooms following algae have been attributed to nutrient limitation, especially nitrogen. For example, two observed genera of oxygenic phototrophs, *Synechococcus* and *Microcystis*, are highly competitive organisms in post‐algal bloom ecosystems, known for their flexibility in utilizing nitrogen sources in low‐nutrient environments (Davis et al., 2009; O'Neil et al., 2012). However, bioavailable nitrogen species experienced little change in the epilimnion: Nitrate and nitrite were consistently depleted (Figure 2d; Table S2), while DON concentrations remained high (240–320 µM; Table S2). Further, both *Picocystis* and Cyanobacteria are capable of utilizing ammonium, which appears to be continuously diffusing into surface waters (Figure 2e). Finally, cyanobacterial success post‐bloom is often attributed to their high surface area to volume ratios that outcompete larger cells for nutrients. This advantage becomes less relevant when compared to Mono Lake's small (<3 µm) *Picocystis*. Although nitrogen limitation has been favored in other systems for explaining cyanobacterial blooms, it seems unlikely to be the main driver in Mono Lake.

We hypothesize that top‐down controls from predation by *Artemia*, rather than bottom‐up factors such as nutrients, were responsible for increased phototrophic niche space. Surveys of Mono Lake's algae have documented that net primary production, as measured by chlorophyll *a* abundance, drops by a factor of two or more during meromixis (Melack et al., 2017). The response of *Artemia* is a single population peak in the spring, rather than two smaller generations in the spring and summer (Figure S3b). This pattern is consistent with our PAR data (Figure S1c), which shows deeper light penetration in spring 2018 compared with spring 2017. Earlier, prolific grazing by *Artemia* would have more efficiently cleared the *Picocystis* bloom, leaving fewer algae for lysis and producing less DOC to support the heterotrophic microbial population. Indeed, our DOC measurements suggest a twofold drop in concentration at 10 m depth from spring 2017 to spring 2018.

It is rare for species competing for the same resources, such as light and nitrogen, to coexist during steady state (Hardin, 1960), although dynamic transitions may create additional niche spaces during succession for organisms to co‐occur (Flöder & Sommer, 1999). In our fall 2018 data, Mono Lake's meromictic epilimnion was characterized by two abundant phototrophs. The codominance of algae and Cyanobacteria has been previously reported in another hypersaline system: At Lake Dziani Dzaha, authors found that *Picocystis salinarum* and *Arthrospira fusiformis* spanned the water column of a monomictic lake (Bernard et al., 2019). They hypothesized that this coexistence related to distinct light adaptations. Indeed, *Picocystis* is specifically adapted for low photon flux and blue‐green wavelengths (~400–500 nm), allowing survival deeper in the epilimnion (Roesler et al., 2002). The most abundant Cyanobacteria in our study belonged to an uncharacterized genus within the non‐diazotrophic marine *Prochlorococcus* and *Synechococcus* clade (Figure 5), but Cyanobacteria in general are often well adapted to high ultraviolet radiation and solar irradiance (Sinha & Hader, 2008), implying greatest success at the top of the photic zone. Future work could increase sampling resolution in the epilimnion to test this possible niche diversification.

Mono Lake's mode of primary productivity shifted drastically as the surface community progressed through microbial succession. If Cyanobacteria continue to populate surface waters, will future bacterial communities respond, switching specialties away from degradation of algal organic matter? Further, some species of *Artemia* are unable to produce sterols and must acquire them from dietary algal sources (D'Abramo et al., 1984; Teshima & Kanazawa, 1971). As most bacteria, including Cyanobacteria, are also incapable of sterol synthesis, replacement of sterol‐producing *Picocystis* in Mono Lake could potentially impact *Artemia* populations, which are critical to the thousands of migratory birds of the Pacific Flyway that flock to Mono Lake annually.

### 4.2 Ecological shifts in the hypolimnion

The water column in spring 2017 was largely oxic to hypoxic (Figure 2c), and the hypolimnion bacterial community resembled that of the epilimnion (Figure 4, Figure S2). With the development of a sharp oxycline, aerobic and aerotolerant populations shoaled. Exceptions included the phototrophic Cyanobacteria and alga *Picocystis*, which maintained substantial abundance in the dark, anoxic hypolimnion throughout the time series (Figure 6b, Figure S1a), an enigma discussed further in Section 4.4. The overall pattern as meromixis developed was a shift from aerobic heterotrophs to anaerobic fermenters and lithotrophic sulfur oxidizers to, eventually, sulfate reducers. In particular, there was a slow rise in obligately anaerobic bacteria, especially the Firmicutes and Bacteroidia. These organisms were likely utilizing ample deep‐water DOC and DON (Figure 2i, Table S2) and/or the degradation products of sinking algal debris and fecal pellets. Branched C_15_ fatty acids also increased over time (Figure 3); Firmicutes genera have been documented to contain 45% C_15_ branched FAs by weight (Chan et al., 1971) and were previously implicated in hypersaline environments with the presence of branched compounds (Jiang et al., 2006). These fermenters in turn would have potentially supplied labile organic substrates for sulfate reducers, as implied by our incubation experiments (Figure 7), which only appeared in significant abundance after prolonged stratification. This transition was surprisingly slow, with the first sulfate reducers appearing three months after hypolimnion anoxia was established, and others appearing after a year of anoxia. We discuss the temporal evolution of sulfur cycling in more detail in Section 4.3.

Interestingly, most of the OTUs observed in the meromictic hypolimnion have been previously identified in Mono Lake sediments (Rojas et al., 2018). To confirm this connection, we characterized the microbial community in sediment core tops in spring 2018 and found persistent symmetry in sediment and hypolimnion bacteria, including genera with fermenting and sulfate‐reducing potential (Figure S5). We hypothesize that the bacterial community developing in Mono Lake's meromictic deep water directly reflects sedimentary species colonizing this expanding, anoxic niche. Previous studies support this claim, finding high resistance in sediment bacterial communities while water‐column communities are disturbed (Diao et al., 2017). These authors postulated that sediments in stratified lakes act as seed banks for anaerobic bacteria and recent reviews have implicated dormancy in seed banks as a mechanism for species resuscitation following environmental changes (Lennon & Jones, 2011). Indeed, spore‐forming Clostridia, which were detected in sediment core tops, were abundant OTUs in the anoxic hypolimnion community of Mono Lake from fall 2017 onward. Taken together, our results for the hypolimnion appear consistent with the idea that sediment seed banks act as a buffer when disturbance alters the chemical composition of a stratified lake.

### 4.3 Temporal evolution of sulfur cycling

Mono Lake supports an active microbial sulfur cycle, with ~110 mM sulfate concentration (Table S2) and significant sulfide porewater fluxes (6.3 mmol m^−2^ day^−1^; Miller et al., 1993). Previous studies have observed different sulfur communities depending on lake stratification: In summer 2016, during the seasonal stratification of a monomictic regime, Stamps et al., (2018) observed abundant sulfide‐oxidizing bacteria in the water column, particularly *Thioalkalivibrio*, but found no evidence of sulfate reducers in the anoxic deep waters. In contrast, multiple studies of the lake under sustained meromictic conditions (>1 year after the onset of stratification) have observed microbial sulfate reduction by diverse deltaproteobacteria at depth (Edwardson & Hollibaugh, 2018; Humayoun et al., 2003; Oremland et al., 2000; Scholten et al., 2005), with sulfide oxidation restricted to the chemocline edge (Edwardson & Hollibaugh, 2017). Our time series provides a detailed view of the ecological transition between these two lake states.

In spring 2017, Mono Lake was monomictic and resembled that described by Stamps et al. (2018), with ~13% of total 16S rRNA reads belonging to *Thioalkalivibrio* (Figure 5). No OTUs representing putative sulfate reducers were observed. By summer 2017, a shallow oxycline had developed, providing a niche with concurrent sulfide and light but minimal oxygen. *Ectothiorhodospira*, an anoxygenic phototroph capable of using reduced sulfur or arsenite as an electron donor, appeared in shallow waters. By fall 2017, Mono Lake had a ~5 m oxycline and hydrogen sulfide was first detected by smell. *Thioalkalivibrio* remained abundant below the chemocline, *Ectothiorhodospira* disappeared, and 16S rRNA sequences for putative sulfate‐reducing organisms appeared for the first time, albeit at low (<1%) relative abundance (Figure S2). The transient appearance of sulfur‐based phototrophy has not been previously reported as an intermediate metabolism, although stable communities of *Ectothiorhodospira* in the hot springs around Paoha island may provide a constant inoculum of this organism to the water column (Kulp et al., 2008; McCann et al., 2017). By spring 2018 and through fall 2018, with sulfide accumulating below the chemocline to ~400 µM (Figure 2f), the sulfur‐cycling community continued to shift. The sulfate reducer *Desulfonatronum* became a dominant organism, with 16S rRNA gene relative abundances of 2%–4% (Figure 5). *Thioalkalivibrio* remained abundant at depth accounting for 2%–4% of reads.

Not until one year after thermal stratification—and at least ten months after anoxia pervaded the hypolimnion—did sulfate reducers reach >1% of the microbial assemblage. This observation of a slow transition from sulfur oxidation to sulfate reduction is in contrast to numerous studies of freshwater lakes, which document rapid expansion of water‐column sulfate reduction following seasonal stratification and anoxia, typically within a few weeks to a month (Cappenberg, 1972; Cook & Schindler, 1983; Hartland et al., 2015; Ingvorsen et al., 1981). Mono Lake's more gradual shift has several implications. First, there is a clear need to consider diversity in the context of time since last turnover in these alkaline, seasonally stratified lakes. Single snapshots of diversity are not necessarily comparable even given a similar lake mixing regime because of hysteresis effects. In Mono Lake, for example, we do not know how long meromixis is required to reach a stable microbial ecosystem. Second, under monomixis, it is unlikely that sulfate reduction would ever become strongly established in Mono Lake's water column. One should therefore be cautious in assuming that seasonally stratified soda lakes will harbor significant populations of sulfate reducers. Third, and more generally, it is unknown whether marine populations of sulfate reducers will adjust quickly like those in freshwater lakes, or very slowly like those in Mono Lake. The implications are significant for generation of toxic concentrations of hydrogen sulfide in seasonal ocean anoxic and hypoxic zones (Thrash et al., 2017; Ulloa et al., 2012).

Limited sulfate reduction in hypersaline lakes has previously been attributed to less favorable energetics of sulfate reduction relative to demands of osmoregulation at high salt stress (Oremland et al., 2000; Oren, 1999). We note that sulfate reducers in Mono Lake multiplied only after the appearance of abundant fermenters, particularly Clostridia and Bacteroidia, in fall 2017 (Figures 5 and 6b). Fermenters typically fill an important ecological function of converting polymeric organic molecules into small organic acids that serve as substrates for anaerobic respiration. We hypothesize that the establishment of sulfate reducers in Mono Lake may be limited more by suitable organic substrates than by the metabolic demands of high salinity. This idea is supported by our microcosm incubations (Figure 7), where sulfide production was significantly enhanced in anoxic Mono Lake water by the addition of lactate and pyruvate—yielding sulfide concentrations up to 3x more than unamended controls. While DOC concentrations in Mono Lake are extremely high (>10 mM), its composition could be highly polymeric and thus largely inaccessible to most sulfate reducers. Future studies could further probe the molecular composition and reactivity of Mono Lake's DOC to study relationships between the available organics and the community of fermenters and sulfur cyclers.

### 4.4 Enigmatic ecophysiology of Picocystis

*Picocystis* sp. strain ML, a close relative of *Picocystis salinarum*, is a small (~3 µm) coccoid green alga that has been an important phytoplankton species in Mono Lake for at least 40 years (Winkler, 1977). While reports through the 1990s list it as subequal in abundance with the diatom *Nitzschia* (Jellison & Melack, 1993; Roesler et al., 2002), recent genetic data suggest a more dominant role for *Picocystis* in Mono Lake, at least during spring through fall (Edwardson & Hollibaugh, 2018; Stamps et al., 2018; this study). For example, in our 16S rRNA data, chloroplast sequences that were not attributable to *Picocystis* comprised <5% relative abundance, over all times and depths. Fatty acid distributions were also extremely similar between lake POC and cultured *Picocystis* (Figure 3). Spring chlorophyll *a* concentrations and turbidity have risen markedly since ~2003 (Collins, 2017) suggesting the shift is relatively recent. The ecological pressures leading to this alga's rise and the subsequent loss of diatoms are unclear but may include the ability of *Picocystis* to thrive in low light at the base of the chemocline throughout extended meromictic events.

*Picocystis* strain ML was originally isolated from a chlorophyll maximum layer near the base of the chemocline during a meromictic phase of the lake in 1997 by Roesler et al. (2002). Subsequent characterization revealed that *Picocystis* strain ML exhibits growth across a remarkable range of salinity (0–260 mg/g), pH (4–12), and light (0.6–12 µmol/m^2^s); this taxon is also resistant to sulfide (100 µM or more), does not require O_2_, and exhibits maximal growth rates of 1.5/d. Its low‐light adaptations are likely due to a diverse suite of carotenoids and ten times higher cellular chlorophyll *a* concentrations compared with other green algae (Roesler et al., 2002). Its ecological niche was therefore interpreted as being throughout the photic zone but especially suited for the base of the chemocline where some sunlight penetrates, ammonia diffuses from below, and low oxygen limits grazing pressure by *Artemia*.

**FIGURE 8:**
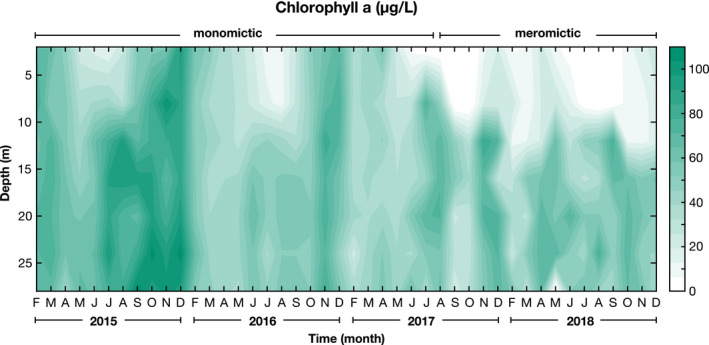
Chlorophyll *a* abundance over time and depth in Mono Lake, CA. Data were redrawn from publicly available (http://www.monobasinresearch.org/onlinereports/) LADWP reports from 2016, 2017, 2018, and 2019. Chlorophyll *a* concentrations were measured by fluorometry after collection at 2, 8, 16, 20, 24, and 28 m water depth. Note that LADWP did not collect data in January of each year. The approximate transition from monomixis to meromixis is also indicated, but it should be noted that these regime shifts are gradual, not sudden

In the two decades since initial characterization, additional monitoring has raised several new questions about *Picocystis*. Chief among them is the observation from Los Angeles Department of Water and Power (LADWP) monthly surveys that *Picocystis* is consistently abundant throughout the hypolimnion, including down to 35 m depth where there must be essentially no photon flux (Collins, 2017, 2018, 2019; Harasick, 2016). We have compiled monthly LADWP data from 2015 to 2019, creating a time series from discrete measurements of chlorophyll *a* fluorescence in the water column (Figure 8). Given that multiple genetic studies revealed *Picocystis* to be the only significant phototroph in present‐day Mono Lake, chlorophyll *a* concentration provides a proxy for *Picocystis* abundance. Our study also found elevated chloroplast reads throughout the hypolimnion (Figure S1; Figure 6b), indicating that *Picocystis* consistently accounted for ~35% of the microbial community at depth. Surprisingly, metatranscriptomics from deep‐water samples recovered mRNA for photosynthetic genes in *Picocystis*, suggesting they were being actively transcribed (Stamps et al., 2018). Taken together, these results beg relatively simple questions regarding this enigmatic alga: Is *Picocystis* alive at the bottom of Mono Lake? And if it is alive, how is it metabolizing and possibly growing?

Despite deep chlorophyll *a* maxima, we do not think it plausible that *Picocystis* is photosynthesizing throughout the hypolimnion. Repeated CTD measurements of PAR find <0.01 µmol/m^2^s photons (the limit of detection by our sensor) at 12–15 m (Figure S1c). Fitting PAR data to an exponential decay, which assumes constant turbidity below the chemocline in accordance with observations during sampling, photon flux would be <10^–5^ µmol/m^2^s below 17 m. Although adaptations to exceedingly low‐light levels are known, for example by an anoxygenic phototrophic *Chlorobium* strain in the Black Sea that survives down to ~10^–4^ µmol/m^2^s (Manske et al., 2005), *Picocystis* has only been tested as low as 0.6 µmol/m^2^s. The fact that photosynthesis genes associated with Photosystem I and II (*psaA*, *psaB*, *psbA*, *psbB*, *psbC*) were being actively transcribed at 25 m depth (Stamps et al., 2018) therefore suggests that those cells were recently at a shallower depth, where photosynthesis was possible, or perhaps that *Picocystis* photosynthetic genes are constitutively expressed. In either case, they appear to be not only alive, but thriving, in the hypolimnion.

We consider two scenarios to explain the depth distribution of *Picocystis*. The first is that *Picocystis* is actively growing at the base of the photic zone and subsequently sinking. Comparison of chlorophyll *a* maxima to our PAR measurements (Figure S1) suggested that these cells are active close to a photon flux of 0.1 µmol/m^2^s, well below the previously tested minimum of 0.6 µmol/m^2^s (Roesler et al., 2002). The depth distribution then requires that those cells must sink rapidly, such that CTD profiles and discrete water samplings record high cell densities throughout the hypolimnion. The near‐simultaneous appearance of Picocystis maxima throughout the hypolimnion implied settling velocities on the order of ~0.5–1 m/day, faster than the ~5–10 cm/day that have been measured for other, notably larger, green algae such as *Scenedesmus* (Choi et al., 2006). However, flocculation into larger aggregate particles could perhaps achieve such rates. In this scenario, cells isolated (or sequenced) from below ~20 m depth should not be interpreted as growing at those depths, but rather as surviving in sinking particles.

A second scenario is that *Picocystis* growth in the hypolimnion is supported partially or entirely by non‐photosynthetic metabolism. In the absence of appreciable exogenous electron acceptors such as oxygen, nitrate, or nitrite, *Picocystis* could perhaps use fermentation to maintain redox homeostasis and conserve energy. The ability to survive transient anoxia via fermentation is widely known in many members of the green algae (Catalanotti et al., 2013). *Chlamydomonas reinhardtii*, the best studied green alga in this regard, has a diverse suite of fermentative pathways and can thrive under anaerobic conditions. When we cross‐referenced *Chlamydamonas* fermentation genes against the recently published draft genome of *Picocystis* sp. strain ML (Junkins et al., 2019), numerous homologous proteins were identified with high confidence (Table S5; Figure S3). However, many of these enzymes also catalyze other cellular functions such as respiration and are alone not evidence of fermentation. Confirmatory laboratory experiments that test the exogenous uptake and subsequent release of carbon compounds by *Picocystis* are required.

Regardless of whether or not *Picocystis* cells are active only at the base of the chemocline or throughout the hypolimnion, it is clear that this ubiquitous alga holds great significance for Mono Lake's biogeochemical cycles and ecology. Numerous questions warrant further investigation, especially in regard to its potential metabolic flexibility, remarkable sulfide tolerance, and transcription of photosynthetic genes in the dark. In addition, our measurements of POC suggested that *Picocystis* strongly fractionates carbon isotopes by up to ~35‰ relative to source DIC, far greater than is expected for algae at such a high pH where [CO_2_(aq)] is extremely low. Eukaryotic strategies for survival in extreme environments are less understood than bacteria and archaea (Weber et al., 2007); investigating *Picocystis* may offer novel insights that span environmental applications.

## 5 CONCLUSIONS

This study captured the microbial community response of hypersaline, alkaline Mono Lake as it transitioned from monomixis to meromixis following climatic perturbation. Patterns of succession in the epilimnion were less predictable than the hypolimnion, presumably due to greater variability in the epilimnion environment. Notably, the epilimnion community shifted from a dominance of heterotrophic to phototrophic genera, with a significant cyanobacterial bloom in fall 2018. The hypolimnion bacterial population gradually shifted toward the composition of Mono Lake's sediment community, strengthening the ecological role of seed banks and dormancy in steering succession in stratified lakes. Specifically, hypolimnion bacterial populations, which began in spring 2017 with aerobic heterotrophs and sulfide oxidizers, were replaced by anaerobic metabolisms such as fermentation by fall 2017 and eventually sulfate reduction in spring 2018. Incubation experiments demonstrated that this late appearance of sulfate reducers was likely due to organic substrate limitation. Hypolimnion communities were consistently dominated by *Picocystis*, which survived meromixis either by photosynthesis at the edge of the chemocline or by a metabolic transition to fermentation. Our investigation across this limnological transition highlights the need to move beyond “snapshots” of microbial communities, probing succession over intervals of environmental perturbations and meaningful climatic change.

## Supporting information

Supplementary Material

## Data Availability

The data that support the findings of this study are available from the corresponding author upon reasonable request. 16S rRNA amplicon sequencing data is available in GenBank under BioProject accession number PRJNA702881.
