# Supplementary material for: Microbial succession and dynamics in meromictic Mono Lake, California
Source: Geobiology. 2021 Feb 25;19(4):376–93. doi: 10.1111/gbi.12437 (PMC8359280; doi:10.1111/gbi.12437)
Supplement: Supplementary file 1 — Supplementary Material [file GBI-19-376-s001.docx]

Supplementary Information

1.0 Methods

1.1 Sediment Sampling

A sediment core ~80cm in length was collected via a hand-held Aquatic Research Instruments universal core head, with 10kg weight and slide hammer. The recovered core had a visible sediment/water interface, and was capped and stored on ice until return to shore (1-3 hours). Overlying water was then removed by siphon, and the core extruded and sectioned in 5cm lengths. The core top used for this study consisted of soupy, dark green mud smelling strongly of sulfide. Sediment sample for sequencing was scooped from the middle of the core and frozen at -20°C until return to Caltech (2 days).

1.2 DNA Extraction Methods

Cell pellets were resuspended in 675 µL 1% CTAB (cetyltrimethylammonium bromide) extraction buffer (100 mM TRIS, 100 mM EDTA, 100 mM Na_2_HPO_4_, 1.5 M NaCl, 1% w/v CTAB) and incubated (37˚C, 30 min) with 50 µL Lysozyme (10 mg/mL). This was followed by incubation (37˚C, 30 min) with 50 µL proteinase K (10mg/mL). Lysis was achieved by incubation (65˚C, 120 min) with 150 µL 10% SDS buffer. Incubation (65˚C, 20 min) with 900 µL phenol/ chloroform/ isoamyl alcohol (25:24:1) was used to separate DNA from cell debris. After benchtop centrifugation (16,000 × *g*, 10 min), the upper aqueous phase was transferred, and trace phenol was removed with 900 µL chloroform/ isoamylalcohol (24:1). DNA was incubated (23˚C, 60 min) with 500 µL isopropanol and precipitated by centrifugation (16,000 × *g*, 15 min). Pellets were washed with ice cold ethanol (70%), centrifuged, decanted, air dried, and stored at -20˚C. 16S amplicons of the V4-V5 region of the 16S rRNA gene were obtained using PCR amplification with 515f and 926r primers (Goffredi et al. 2020). The protocol followed a 2 min initial denaturation at 98˚C, 28 cycles of 10 s at 98˚C, 20 s at 54˚C, 20 s at 72˚C, and a final elongation for 2 min at 72˚C. Product size was verified using a 1.5% agarose gel and 100 bp ladder (Fisher Scientific).

1.3 Modified Cline Assay for Sulfide

The Cline assay for hydrogen sulfide had to be modified for Mono Lake water because the high DIC content causes precipitation of large amounts of zinc carbonate when samples are ‘fixed’ using zinc acetate. Precipitated zinc sulfide samples were first rinsed with ultrapure water three times, after which water was added to reach a final volume of 10 mL. Samples were shaken vigorously to resuspend the precipitate and 100 µL aliquots were taken and added into 20 µL of a 1:1 mixture of 11.5 mM N,N-dimethyl-p-phenylenediamine dihydrochloride (DPDD) in 6 N HCl and 30 mM FeCl_3_. The resulting blue solution was measured for absorbance at 670 nm with a Tecan Sunrise-Basic microplate reader with Magellan software (version 5.0.3.5) at Caltech. Standard curves were generated with 50 µM to 2 mM solutions of zinc sulfide (10 µm) prepared with sulfide-free Mono Lake water. Samples were diluted to be measured within the standard curve’s range.

1.4 DON Measurement Details

In a pre-combusted 4 mL glass vial, 0.1 mL of Mono Lake water was combined with 1 mL persulfate oxidizing reagent (1 g recrystallized low-N K_2_S_2_O_8_ and ACS grade 1.5 g NaOH in 100 mL ultrapure water) and autoclaved for 1 hr. DON concentration was calculated by subtracting the concentrations of nitrate and ammonium/ammonia from the concentration of TDN. The nitrogen concentration of blanks (persulfate oxidizing reagent) were measured to be <0.3 μM.

1.5 Fatty Acid Extraction and Measurement

POC filters were placed in Teflon tubes with 20 mL dichloromethane (DCM) and methanol (9:1) in a Microwave Accelerated Reaction System 5 (CEM Instruments; 8 min ramp to 100˚C, hold for 15 min with stirring). Extracts were saponified in 0.5 M NaOH (70˚C, 4 hrs), adjusted to pH 1.5 then extracted into methyl tert-butyl ether (MTBE). Fatty acids were purified via solid phase extraction using 0.5 g of Sepra NH2 stationary phase (Phenomenex; 50 µm, 65 Å) and eluted in 2.5% formic acid in DCM (8 mL), Collected fatty acids were then converted to fatty acid methyl esters (FAMEs) by reaction (70°C, 10 min) in DCM (3 mL) and 14% BF_3_ in methanol (100 µL). FAMEs were separated on a Thermo Scientific Trace GC equipped with a 30 m x 0.25 mm ZB-5MS capillary column (Phenomenex Zebron; 0.25 µm film thickness) using helium as the carrier gas (0.8 mL/min). Detection was with a Thermo Scientific DSQII mass spectrometer, with electron-impact ionization source operated at 70 eV and 250˚C and 50-800 Da full scan. Samples were injected in splitless mode with a PTV injector. The GC oven was programmed at 100˚C (1 min), heated to 140˚C (20˚C/min), to 250˚C (3˚C/min), to 310˚C (20˚C/min) and held at 310˚C (10 min). Samples were identified using mass spectral comparisons with the NIST 200x database and laboratory standards. Compounds were quantified by comparing TIC peak areas to an internal standard (palmitic acid isobutyl ester) assuming equal response factors.

1.6 MATLAB Heatmap Code

16S Amplicon Data Visualization

%open16S data and save as new variables called above1percent and below1percent

maxbelow=max(below1percent(:));

minbelow=min(below1percent(:));

maxabove=max(above1percent(:));

minabove=min(above1percent(:));

f1 = figure;

color1 = [255 255 255]/255; %color for lowest value (0.0), white

color2 = [66 147 195]/255;%color for middle value (1.0), blue

color3 = [213 96 77]/255;%color for highest value (maximum of above1percent), black

ntotal = 1000;%total # of colors in gradient

midcolorval = 1.0;%value for middle color

ncolors1 = ntotal*midcolorval/maxabove;

ncolors2 = ntotal-ncolors1;

map1 = [linspace(color1(1),color2(1),ncolors1),linspace(color2(1),color3(1),ncolors2);

linspace(color1(2),color2(2),ncolors1),linspace(color2(2),color3(2),ncolors2);

linspace(color1(3),color2(3),ncolors1),linspace(color2(3),color3(3),ncolors2)]';

h=heatmap(above1percent,'ColorMap',map1);

f2 = figure;

colormaxbelow = maxbelow/midcolorval*(color2-color1)+color1; %Color for 0.86 value

ncolorsbelow = ntotal;

map2 = [linspace(color1(1),colormaxbelow(1),ncolorsbelow);

linspace(color1(2),colormaxbelow(2),ncolorsbelow);

linspace(color1(3),colormaxbelow(3),ncolorsbelow)]';

h=heatmap(below1percent,'ColorMap',map2);

2.0 Results

2.1 Inorganic Chemistry (DIC and Major Cations)

Major element abundances were measured only in spring 2017: concentrations of K^+^ (1.18 – 1.24 ppt), Mg^2+^ (20.10 – 21.51 ppm), Ca^2+^ (11.55 – 12.87 ppm), Si^4+^ (9.34 – 9.83 ppm), Sr^2+^ (6.54 – 7.46 ppm), Li^2+^ (2.08 – 3.64 ppm), Fe^2+^ (39.82 – 42.08 ppb) and Mn^2+^ (11.03 – 13.63 ppb) were consistent with previous reports (Gray et al. 2008; Garrels & MacKenzie, 1967). DIC concentrations, also measured only in spring 2017, spanned 384 – 396 mM, agreeing with published values for Mono Lake at the same approximate lake level (Oremland, Stolz, & Hollibaugh, 2004). The δ^13^C_VPDB_ values of DIC were also measured, averaging 2.2 ± 0.2‰, matching previous studies by Li & Ku (1996) who reported the δ^13^C_PDB_ as 2‰ for Mono Lake.

2.2 Picocystis Fatty Acid Composition

The FA composition of *Picocystis* has not been previously described. *Picocystis* synthesized a variety of FAs. Palmitic acid (C_16_) was the most abundant—53% of the total signal. Monounsaturated, even-numbered FAs, including one isomer of hexadecenoic acid (16:1) and three isomers of octadecenoic acid (18:1) were also significant, accounting for a further 26% of the FA profile. Of the monounsaturated FAs, 16:1 was most abundant. Polyunsaturated fatty acids, with two or more double bonds, were 12% of normalized FA abundance and comprised 16:3, 18:2, 20:4, 20:3, and 20:2. Anteiso 16:0 was also detected in significant quantities, accounting for 4% of *Picocystis* fatty acids. Interestingly, iso and anteiso C_17_ FA were also identified, but at much lower levels (<1%). A series of even fatty acids ranging from C_14_ to C_18_ with a hydroxyl group at C-3 were also found in low abundance (<1%). Similar β-hydroxy FA’s have been previously reported in green algae (Matsumoto & Nagashima, 1984).

2.3 Species Richness, Evenness, and Shannon Diversity

Species richness—a measure of the number of OTUs—was higher in the hypolimnion, where OTUs increased after prolonged stratification. Epilimnion species richness was more variable, peaking with summer stratification and stabilizing under meromictic conditions. Shannon diversity ranged from 5.10 to 6.52, values similar to other freshwater stratified lakes (Roger et al. 2016). Like species richness, Shannon diversity was higher in the hypolimnion than the epilimnion. Evenness, however, was similar across these two depth regimes until spring 2018, when evenness dropped to a low of 0.80 in the epilimnion.

2.4 16S rRNA Gene Amplicon Taxa Abundances

Eukaryotes

98.7% of chloroplast sequences were assigned to the dominant phototrophic alga, *Picocystis*, while 0.9% belonged to a *Dictyochophyceae* genus and 0.2% to a *Chrysophyceae* genus. Only trace (<0.1%) chloroplast reads were assigned to Bacillariophyceae (diatoms).

Epilimnion Bacteria

In spring 2017, Mono Lake was monomictic and the epilimnion microbial community was characterized by the phototrophic alga *Picocystis* (~20% relative abundance) and aerobic or aerotolerant heterotrophic bacteria (~40% relative abundance) within Alphaproteobacteria, Gammaproteobacteria, Cytophagia, Actinobacteria, Bacteroidia, and Flavobacteriia. Notable Alphaproteobacteria detected included members of the photoheterotrophic genera *Roseovarius* and *Rhodobaca*, which were first isolated from hypersaline lakes (Labrenz et al., 1999; Milford et al. 2000). Heterotrophic genera of the Gammaproteobacteria, including *Methylonatrum* and *Spiribacter*, were also abundant. Characterized members of the *Spiribacter* genus degrade osmolytes like glycerol, secreted in abundance by algae under salt stress (Lopez-Perez et al., 2013) while *Methylonatrum* isolates oxidize numerous C_1_ and C_2_ compounds like formate, ethanol, methanol, and dimethylamine (Rua & Thompson, 2014). Notable Bacteroidetes genera included the cryophilic heterotrophs *Psychroflexus*, and the potential polysaccharide degraders Cyclobacteriaceae. The type species of the abundant Actinobacteria, *Nitriliruptor*, was originally isolated from a soda lake (Sorokin et al. 2009). Finally, *Spirochaeta*, a genus of facultatively anaerobic chemoheterotrophs and the Verrucomicrobia (genus *Opitutus*) known to degrade complex polysaccharides such as xylan and pectin (Rodrigues & Isanapong, 2014) were also identified. In fall 2017 there was an increase in unclassified sequences. We can say little about this unknown community, but due to coincident changes in hydrology, these OTUs may in part reflect organisms delivered directly from fresh water. This abundance of unknown taxa remained throughout spring 2018 as *Picocystis* bloomed.

Hypolimnion Bacteria

Other likely heterotrophic bacteria were identified in the hypolimnion, including *Methylomicrobium*—an obligate methane or methanol oxidizer (Kalyuzhnaya et al. 2008). Bacteroidia were dominated by an unknown genus in the ML635J*-*40 order, previously identified in a clone library of Mono Lake deep water. The majority of Firmicute OTUs belonged to Clostridia, a class of predominantly spore-forming anaerobic fermenters. *Syntrophomonadaceae*, short-chain (C_4_+) carboxylic acid degraders (Sobieraj & Boone, 2006) and *Ruminococcus*, carbohydrate fermenters, were the most abundant Firmicutes (Figure 5). Bacteroidia were dominated by an unknown genus in the ML635J*-*40 order, previously identified in a clone library of Mono Lake deep water. The majority of Firmicute OTUs belonged to Clostridia, a class of predominantly spore-forming anaerobic fermenters. *Syntrophomonadaceae*, short-chain (C_4_+) carboxylic acid degraders (Sobieraj & Boone, 2006) and *Ruminococcus*, carbohydrate fermenters, were the most abundant Firmicutes (Figure 7). Many other genera also increased in the hypolimnion at this time, with concurrent rises in both species richness and Shannon diversity (Table S4, Figure 6). While no single taxon dominated, the relative abundance of all Firmicutes was significant, accounting for almost 10% of the microbial community (Figures 8b, S2). Detected (> 0.1% relative abundance) members of Deltaproteobacteria included the sulfate reducing bacteria *Desulfonatronum, Desulfonatronovibrio,* and two uncharacterized genera from *Desulfobacteraceae* and *Desulfohalobiaceae* families. Notably, the *Contubernalis* genus, containing known syntrophic partners to *Desulfobacteraceae* and *Desulfohalobiaceae* was also detected. This anaerobic alkaliphile was previously isolated from soda lakes (Zhilina et al. 2005; Sorokin et al., 2014). Sulfur reducers capable of using electron acceptors other than sulfate, such as thiosulfate or elemental sulfur, were also detected, including the spore-forming firmicute *Dethiobacter* as well as the Deltaproteobacteria *Desulfuromusa.* Several lithotrophic organisms with sulfur-oxidizing potential were identified. The most abundant sulfur oxidizer, *Thioalkalivibrio*, a chemolithoautotrophic halophile previously isolated from Mono Lake (Sorokin et al., 2002), accounted for up to ~10% of the microbial community at depth (Figures 4, 5). Other sulfur oxidizers were detected including *Thiomicrospira* and *Ectothiorhodospira*, also in the Gammaproteobacteria. Many of these sulfur cyclers persisted in the hypolimnion into summer 2017 as Mono Lake entered thermal stratification. Cyanobacteria that bloomed in the last time point were also surprisingly abundant in hypolimnion samples, although reasons for their appearance are unclear and could be due to sinking particles following their bloom or their transient survival at the edge of the chemocline.

3.0: Discussion

*Picocystis*’ Enigmatic Carbon Isotope Fractionation

We observed remarkably large carbon isotope offsets between particulate organic matter and lake DIC. Mono Lake POM, which is dominantly algal biomass in deep waters (Figure 3), yielded an average δ^13^C value of -31.6 ± 0.3‰ in the hypolimnion, which we interpret as *Picocystis*’ bulk biomass value. δ^13^C measurements of DIC yielded an average value of 2.2 ± 0.2‰ across depths. These offsets imply a large carbon isotope fractionation associated with photosynthesis, on the order of ~34‰ relative to source DIC and ~27-29‰ relative to dissolved CO_2_. This is unexpected because larger isotope fractionations (up to ~25‰ relative to dissolved CO_2_) are typically correlated with high concentrations of dissolved CO_2_, which is negligible in alkaline Mono Lake (Popp et al. 1998; Wilkes & Pearson, 2019). Further, if *Picocystis* is instead using bicarbonate as its primary inorganic carbon source, we would expect smaller net fractionations because bicarbonate is enriched in ^13^C compared to CO_2_ and the carbon isotope fractionation of RuBisCO (Ribulose-1,5-bisphosphate carboxylase/oxygenase) is minimized due to closed-system behavior (Zeebe & Wolf-Gladrow 2001). From investigating the *Picocystis* sp. strain ML genome against known genes in *Chlamydamonas reinhardtii*, *Picocystis* indeed appears to have machinery to take up bicarbonate, including putative transporters and carbonic anhydrases that comprise a carbon concentrating mechanism (Atkinson et al. 2016; Table S5). However, the detailed pathways of carbon acquisition and fixation in *Picocystis* and their associated isotopic consequences are unknown and – like much about this alga – beg further investigation.

*Table S1 | Information regarding which samples were collected for each time point, as well as date of sampling, water depths sampled, and method of water collection.*

|  | Spring 2017 | Summer 2017 | Fall  2017 | Spring 2018 | Summer 2018 | Fall  2018 |
| --- | --- | --- | --- | --- | --- | --- |
| Date | 5/23/17 | 6/22/17 | 9/19/17 | 5/8/18 | 6/13/18 | 10/9/18 |
| Sample Depths (m) | 0.5, 10, 20, 25, 30 | 0.5, 10, 15, 20, 27 | 0.5, 5, 10, 15, 20, 24 | 0.5, 5, 10, 12, 17, 20, 25, 35 | 5, 12, 15, 17, 30 | 0.5, 5, 12, 15, 17, 20, 30, 35 |
| Collection Method | Niskin | Niskin | Pump | Niskin | Niskin | Pump |
| CTD Cast | ✓ | ✓ | ✓ | ✓ | ✓ | ✓ |
| DNA | ✓ | ✓ | ✓ | ✓ |  | ✓ |
| Nutrients | ✓ |  | ✓ | ✓ | ✓ | ✓ |
| POC |  |  | ✓ | ✓ | ✓ | ✓ |
| δ^13^C POC |  |  | ✓ | ✓ | ✓ | ✓ |
| DOC | ✓ | ✓ | ✓ | ✓ | ✓ | ✓ |
| DON |  |  | ✓ |  | ✓ |  |
| DIC | ✓ |  |  |  |  |  |
| δ^13^C DIC | ✓ |  |  |  |  |  |
| Sulfate | ✓ |  |  |  |  | ✓ |
| Sulfide |  |  |  | ✓ | ✓ | ✓ |
| Incubations |  |  |  |  |  | ✓ |
| Fatty Acids |  |  | ✓ | ✓ | ✓ | ✓ |
| Major Elements | ✓ |  |  |  |  |  |

*Table S2 | Measured chemical compositions of Mono Lake throughout the time series. N.S. denotes “not sampled” and B.D.L. denotes “below detection limit.” Values reported are ranges found within the water column for each time point. When depth trends were apparent, they were plotted and presented in the main text.*

|  | Spring 2017 | Summer 2017 | Fall  2017 | Spring 2018 | Summer 2018 | Fall  2018 |
| --- | --- | --- | --- | --- | --- | --- |
| K^+^  (ppt) | 1.18 –  1.24 | N.S. | N.S. | N.S. | N.S. | N.S. |
| Mg^2+^  (ppm) | 20.10 – 21.51 | N.S. | N.S. | N.S. | N.S. | N.S. |
| Ca^2+^  (ppm) | 11.55 – 12.87 | N.S. | N.S. | N.S. | N.S. | N.S. |
| Si^4+^  (ppm) | 9.34 –  9.83 | N.S. | N.S. | N.S. | N.S. | N.S. |
| Sr^2+^  (ppm) | 6.54 –  7.46 | N.S. | N.S. | N.S. | N.S. | N.S. |
| Li^2+^  (ppm) | 2.08 –  3.64 | N.S. | N.S. | N.S. | N.S. | N.S. |
| Fe^2+^  (ppb) | 39.82 – 42.08 | N.S. | N.S. | N.S. | N.S. | N.S. |
| Mn^2+^  (ppb) | 11.03 – 13.63 | N.S. | N.S. | N.S. | N.S. | N.S. |
| Nitrite  (µM) | 0.05 –  0.10 | N.S. | 0.10 –  0.20 | B.D.L. | 0.00 –  0.16 | 0.02 –  0.29 |
| Nitrate  (µM) | 0.05 –  1.45 | N.S. | 0.85 –  1.35 | 0.05 –  0.12 | 0.00 –  0.07 | 0.00 –  0.03 |
| Silica  (µM) | 514.50 – 535.50 | N.S. | 455.50 – 559.50 | 482.00 – 597.00 | 446.00 – 625.00 | 489.00 – 708.00 |
| Phosphate  (µM) | N.S. | N.S. | 643.37 – 779.72 | 573.00 – 754.00 | 641.00 – 712.00 | 618.00 – 774.00 |
| Ammonium (µM) | 0.68 –  3.49 | N.S. | 1.45 –  44.80 | 0.24 – 103.10 | 0.18 – 102.00 | 0.33 – 141.00 |
| Sulfide  (µM) | N.S. | N.S. | N.S. | 299 –  464 | 155 –  407 | 152 –  391 |
| Sulfate  (mM) | 98.31 – 111.00 | N.S. | N.S. | N.S. | N.S. | 101.70 – 115.20 |
| DIC  (mM) | 384 –  396 | N.S. | N.S. | N.S. | N.S. | N.S. |
| δ^13^C DIC (‰) | 2.1 –  2.4 | N.S. | N.S. | N.S. | N.S. | N.S. |
| DOC  (mM) | 9.79 – 21.00 | 9.13 – 21.31 | 8.13 –  10.01 | 10.81 –  14.13 | 10.73 –  14.30 | 10.87 –  14.62 |
| POC  (mM) | N.S. | N.S. | 1.71 –  1.78 | 0.92 –  1.60 | 0.72 –  1.70 | 0.50 –  2.18 |
| δ^13^C POC (‰) | N.S. | N.S. | -32.2 –  -30.2 | -32.1 –  -31.5 | -31.9 –  -31.3 | -31.5 –  -30.6 |
| DON  (mM) | N.S. | N.S. | 0.24 –  0.34 | N.S. | 0.28 –  0.32 | N.S. |

*Table S3 | Fatty acid concentrations per sample, given in ng/mL, organized by time point and depth. Quantification was obtained by use of an internal standard. C21-C30 saturated fatty acids were assumed to be terrestrially sourced and were removed before normalizations for Figure 5.*

|  |  | Present in Picocystis Culture | | | | | Not Identified in Picocystis Culture | | | |
| --- | --- | --- | --- | --- | --- | --- | --- | --- | --- | --- |
| Time Point | **Depth (m)** | **C14-C20 Saturated** | **C16-C18 Even Mono-unsaturated** | **C18-C20 Even Poly-unsaturated** | **C14-C18 Even Hydroxyl** | **C16 -C17 Branched** | **C17 Odd Branched** | **C14-C18 Even Mono-unsaturated** | **C15 Odd Branched** | **C21-C30 Saturated** |
| Fall 2017 | 5 | 34.36 | 7.65 | 2.21 | 0.35 | 0.19 | 0.17 | 0.88 | 0.10 | 1.30 |
|  | 15 | 141.54 | 22.85 | 13.39 | 1.43 | 0.21 | 0.71 | 4.26 | 0.32 | 10.46 |
| Spring 2018 | 0.5 | 133.90 | 18.83 | 2.23 | 0.16 | 0.14 | 0.22 | 1.19 | 0.12 | 1.93 |
|  | 20 | 91.81 | 9.92 | 1.57 | 0.80 | 0.19 | 0.28 | 2.15 | 0.14 | 6.94 |
|  | 25 | 91.81 | 7.90 | 0.97 | 0.14 | 0.08 | 0.19 | 1.17 | 0.18 | 2.90 |
| Summer 2018 | 5 | 20.46 | 4.66 | 3.83 | 0.00 | 0.00 | 0.09 | 0.23 | 0.05 | 0.81 |
|  | 12 | 41.00 | 4.04 | 0.15 | 0.16 | 0.07 | 0.15 | 0.71 | 0.01 | 2.36 |
|  | 15 | 53.08 | 7.24 | 0.30 | 0.17 | 0.20 | 0.23 | 2.22 | 0.03 | 3.35 |
|  | 17 | 14.13 | 3.17 | 0.53 | 0.24 | 0.08 | 0.13 | 1.11 | 0.05 | 2.49 |
|  | 30 | 12.97 | 1.33 | 0.20 | 0.17 | 0.03 | 0.03 | 0.36 | 0.02 | 0.78 |
| Fall 2018 | 5 | 29.90 | 7.26 | 0.51 | 0.15 | 0.09 | 0.20 | 0.62 | 0.07 | 8.58 |
|  | 30 | 49.83 | 6.42 | 1.10 | 0.36 | 0.29 | 0.23 | 2.48 | 0.13 | 2.83 |
|  | 35 | 39.90 | 7.24 | 1.64 | 0.38 | 0.42 | 0.34 | 3.49 | 0.14 | 4.65 |

*Table S4 | Richness, Shannon Diversity, and Evenness for the 32 samples from the Mono Lake water column. The samples were rarified to 1370, the least number of OTUs in any sample after removal of chloroplasts (Picocystis salinarum) and fully unassigned sequences.*

| Season and Year | Depth (m) | Richness | Shannon Diversity | Evenness |
| --- | --- | --- | --- | --- |
| Spring 2017 | 0.5 | 618 | 5.382 | 0.837 |
|  | 10 | 727 | 5.807 | 0.881 |
|  | 20 | 689 | 5.576 | 0.853 |
|  | 25 | 694 | 5.581 | 0.853 |
|  | 30 | 702 | 5.609 | 0.856 |
| Summer 2017 | 0.5 | 761 | 5.890 | 0.888 |
|  | 10 | 770 | 6.081 | 0.915 |
|  | 15 | 614 | 5.874 | 0.915 |
|  | 20 | 626 | 5.218 | 0.810 |
|  | 27 | 596 | 5.119 | 0.801 |
| Fall 2017 | 0.5 | 790 | 6.034 | 0.904 |
|  | 5 | 689 | 5.808 | 0.889 |
|  | 10 | 896 | 6.348 | 0.934 |
|  | 15 | 862 | 6.260 | 0.926 |
|  | 20 | 817 | 6.08 | 0.91 |
|  | 24 | 822 | 6.096 | 0.908 |
| Spring 2018 | 0.5 | 548 | 5.839 | 0.926 |
|  | 5 | 472 | 5.672 | 0.921 |
|  | 10 | 562 | 5.870 | 0.927 |
|  | 12 | 609 | 6.141 | 0.958 |
|  | 17 | 839 | 6.231 | 0.926 |
|  | 20 | 919 | 6.497 | 0.952 |
|  | 25 | 919 | 6.479 | 0.950 |
|  | 35 | 948 | 6.517 | 0.951 |
| Fall 2018 | 0.5 | 658 | 5.234 | 0.807 |
|  | 5 | 633 | 5.109 | 0.792 |
|  | 12 | 625 | 5.137 | 0.798 |
|  | 15 | 825 | 6.013 | 0.895 |
|  | 17 | 836 | 6.069 | 0.902 |
|  | 20 | 834 | 6.091 | 0.906 |
|  | 30 | 829 | 6.108 | 0.909 |

*Table S5 | Genes identified in Chlamydomonas for potential fermentation pathways and carbon concentration mechanisms, checked against the draft genome for Picocystis sp. ML. E-value cutoffs of E-6 were used. E values are calculated based on the alignment score and represent the expected number of hits with that alignment score or better. Thus, lower E values indicative of more statistically robust results.*

| Function | Gene | Gene Name | Accession Number | In Pico ML? | % Identity | E Value |
| --- | --- | --- | --- | --- | --- | --- |
| Fermentation | ACK1 | Acetate kinase | XP_001694505.1 | **No** | N.A. | N.A. |
|  | ACK2 |  | XP_001691682.1 | **No** | N.A. | N.A. |
|  | ACS1 | Acetyl co-A synthase | XP_001700210.1 | **Yes** | 62.25 | 0 |
|  | ACS2 |  | XP_001700230.1 | **Yes** | 65.56 | 0 |
|  | ACS3 |  | XP_001702039.1 | **Yes** | 55.36 | E-28 |
|  | ADH1 | Alcohol/ acetaldehyde dehydrogenase | XP_001703585.1 | **Yes** | 51.82 | 0 |
|  | AAT1 | Alanine aminotransferase | XP_001698518.1 | **Yes** | 48.34 | E-142 |
|  | AAT2 |  | XP_001695350.1 | **Yes** | 61.59 | E-111 |
|  | ALD1 | Aldehyde dehydrogenase | XP_001696928.1 | **Yes** | 58.30 | 0 |
|  | FDX5 | Ferredoxin | XP_001690910.1 | **Yes** | 38.71 | E-7 |
|  | FUM1 | Fumarase hydratase | XP_001696634.1 | **No** | N.A. | N.A. |
|  | FUM2 |  | XP_001689951.1 | **Yes** | 62.23 | E-179 |
|  | GK | Glycerol kinase | XP_001692205.1 | **Yes** | 61.54 | E-61 |
|  | GPD1 | Glycerol-3-phosphate dehydrogenase | XP_001693544.1 | **Yes** | 45.65 | E-86 |
|  | GPP1 | Glycerol 3-phosphate phosphatase | XP_001689796.1 | **Yes** | 52.13 | E-90 |
|  | GPP2 |  | XP_001689798.1 | **Yes** | 51.56 | E-91a |
|  | HYD1 | Iron hydrogenase | XP_001693376.1 | **Yes** | 28.57 | E-39 |
|  | HYD2 |  | XP_001694503.1 | **Yes** | 31.98 | E-25 |
|  | LDH | Lactate dehydrogenase | XP_001700756.1 | **No** | N.A. | N.A. |
|  | MDH1 | Malate dehydrogenase | XP_001693118.1 | **Yes** | 61.86 | E-123 |
|  | MDH2 |  | XP_001702586.1 | **Yes** | 59.34 | E-107 |
|  | MDH3 |  | XP_001694886.1 | **Yes** | 67.55 | E-92 |
|  | MDH4 |  | XP_001703167.1 | **Yes** | 74.60 | E-150 |
|  | MDH5 |  | XP_001696786.1 | **Yes** | 41.57 | E-51 |
|  | MME1 | Malic enzyme | XP_001696240.1 | **Yes** | 62.23 | E-99 |
|  | MME2 |  | XP_001692778.1 | **Yes** | 43.44 | E-92 |
|  | MME3 |  | XP_001692779.1 | **Yes** | 41.99 | E-94 |
|  | MME4 |  | XP_001692684.1 | **Yes** | 62.00 | E-76 |
|  | MME5 |  | XP_001689967.1 | **Yes** | 35.53 | E-71 |
|  | MME6 |  | XP_001696415.1 | **Yes** | 34.82 | E-81 |
|  | PAT1 | Phosphate acetyltransferase | XP_001700210.1 | **No** | N.A. | N.A. |
|  | PAT2 |  | XP_001700210.1 | **No** | N.A. | N.A. |
|  | PDC3 | Pyruvate decarboxylase | XP_001703530.1 | **Yes** | 29.00 | E-50 |
|  | PEPC | Phosphoenol-pyruvate carboxylase | XP_001695817.1 | **Yes** | 54.63 | E-128 |
|  | PFL1 | Pyruvate formate lyase | XP_001689719.1 | **No** | N.A. | N.A. |
|  | PRF1 | Pyruvate ferredoxin oxidoreductase | XP_001701208.1 | **No** | N.A. | N.A. |
|  | PYC | Pyruvate carboxylase | XP_001696348.1 | **Yes** | 50.30 | E-177 |
|  | PYK1 | Pyruvate kinase | XP_001693008.1 | **Yes** | 77.28 | 0 |
| Carbon Concentration Mechanism | HLA3 | ABC transporter | XP_001700040.1 | **Yes** | 33.95 | E-84 |
|  | LCI1 | Low CO_2_ inducible membrane protein | XP_001703387.1 | **No** | N.A. | N.A. |
|  | LCI2 |  | XP_001695920.1 | **Yes** | 42.65 | E-11 |
|  | LCI3 |  | XP_001697649.1 | **No** | N.A. | N.A. |
|  | LCI5 |  | XP_001690584.1 | **No** | N.A. | N.A. |
|  | LCI6 |  | XP_001694024.1 | **No** | N.A. | N.A. |
|  | LCI9 |  | XP_001694765.1 | **No** | N.A. | N.A. |
|  | LCI11 |  | XP_001697963.1 | **Yes** | 43.56 | E-13 |
|  | LCI13 |  | XP_001698750.1 | **No** | N.A. | N.A. |
|  | LCI21 |  | XP_001692958.1 | **No** | N.A. | N.A. |
|  | LCI22 |  | XP_001702373.1 | **No** | N.A. | N.A. |
|  | LCI23 |  | XP_001695444.1 | **No** | N.A. | N.A. |
|  | LCI24 |  | XP_001703408.1 | **No** | N.A. | N.A. |
|  | LCI30 |  | XP_001690835.1 | **Yes** | 78.46 | E-136 |
|  | LCI31 |  | XP_001691792.1 | **Yes** | 33.74 | E-7 |
|  | LCI33 |  | XP_001701266.1 | **No** | N.A. | N.A. |
|  | LCI34 |  | XP_001691880.1 | **No** | N.A. | N.A. |
|  | LCI36 |  | XP_001696214.1 | **Yes** | 35.08 | E-33 |
|  | LCIB |  | XP_001698344.1 | **Yes** | 48.05 | E-90 |
|  | LCIC |  | AB168094.1 | **Yes** | 40.00 | E-97 |
|  | CCP1 | Low CO_2_ inducible chloroplast envelope protein | XP_001692197.1 | **Yes** | 28.57 | E-35 |
|  | CCP2 |  | XP_001692288.1 | **Yes** | 29.55 | E-37 |
|  | CAG1 | Carbonic Anhydrase | XP_001703237.1 | **Yes** | 43.79 | E-41 |
|  | CAG2 |  | XP_001701594.1 | **Yes** | 42.97 | E-37 |
|  | CAG3 |  | XP_001696746.1 | **Yes** | 60.82 | E-60 |
|  | CAH1 |  | XP_001692291.1 | **No** | N.A. | N.A. |
|  | CAH2 |  | XP_001692290.1 | **No** | N.A. | N.A. |
|  | CAH4 |  | XP_001696003.1 | **No** | N.A. | N.A. |
|  | CAH5 |  | XP_001700770.1 | **No** | N.A. | N.A. |
|  | CAH6 |  | XP_001703176.1 | **No** | N.A. | N.A. |
|  | CAH7 |  | XP_001699151.1 | **No** | N.A. | N.A. |
|  | CAH8 |  | XP_001697606.1 | **No** | N.A. | N.A. |
|  | CAH9 |  | XP_001700909.1 | **No** | N.A. | N.A. |

*Figure S1 | Parameters relevant to* Picocystis*: a) 16S rRNA gene amplicons assigned to chloroplasts, interpreted as* Picocystis *b) chlorophyll a concentrations for the same months as our sampling, redrawn from 2018 and 2019 LADWP Compliance Reports and c) PAR (photosynthetically active radiation, which can be interpreted as photon flux, as measured by CTD. For each panel data is plotted as Spring 2017 (red circles), Summer 2017 (orange inverted triangles), Fall 2017 (yellow squares), Spring 2018 (green triangles), Summer 2018 (blue diamonds) and Fall 2018 (purple pentagons).*

*Figure S2 | Heatmap of 16S rRNA relative abundance for genera between 0.1 – 1.0% at Station 6. This figure uses the same color scale and ordering of time and depth points as in Figure 7.*

**

*Figure S3 |* Artemia *population data for a) adults and b) nauplii and juveniles, redrawn from publicly available (http://www.monobasinresearch.org/onlinereports/) LADWP reports from 2018 and 2019. Average population is seen per meter squared across the lake’s 12 monitored stations in seen in panel. Instar abundance, spanning nauplii and juveniles, was averaged for each month for meromictic and monomictic years when such monthly data was available (1987-present).*

*Figure S4 | Potential algal fermentation pathways in the model green alga* Chlamydomonas reinhardtii*, redrawn from Catalanotti et al. 2013. Genes colored in black are those with homologous proteins (E value < E-6, Table S5) observed in the* Picocystis *sp. ML draft genome. In gray are genes present in* Chlamydomonas *but not* Picocystis*.*

**

*Figure S5 | Heatmap of 16S rRNA gene amplicons (that at one point reached >0.1% relative abundance) of water column samples and core top sediment samples from Spring 2018.*

References:

Atkinson, N., Feike, D., Mackinder, L.C.M., Meyer, M.T., Griffiths, H., Jonikas, M.C., Smith, A.M., & McCormick, A.J. (2016). Introducing an algal carbon-concentrating mechanism into higher plants: location and incorporation of key components. Plant Biotechnology Journal, 14, 1302-1315.

Garrels, R.M., MacKenzie, F.T. (1967). Origin of the chemical compositions of some springs and lakes. Equilibrium Concepts in Natural Water Systems Advances in Chemistry, 67, 222-242.

Goffredi, S.K., Tilic, E., Mullin, S.W., Dawson, K.S., Keller, A., Lee, R.W., Wu, F., Levin, L.A., Rouse, G.W., Cordes, E.E., Orphan, V.J. (2020). Methanotrophic bacterial symbionts fuel dense populations of deep-sea feather duster worms (Sabellida, Annelida) and extend the spatial influence of methane seepage. Science Advances, 6, 1-12.

Gray, W., Glazner, A.F., Coleman, D.S., & Bartley, J.M. (2008). Long-term geochemical variability of the Late Cretaceous Tuolumne Intrusive Suite, central Sierra Nevada, California. Geological Society London, 304, 183-201.

Kalyuzhnaya, M.G., Khmelenina, V., Eshinimaev, B., Sorokin, D., Fuse, H., Lidstrom, M., & Trotsenko, Y. (2008). Classification of halo(alkali)philic and halo(alkali)tolerant methanotrophs provisionally assigned to the genera Methylomicrobium and Methylobacter and emended description of the genus Methylomicrobium. ISME Journal, 58, 591-596.

Labrenz, M., Collins, M.D., Lawson, P.A., Tindall, B.J., Schumann, P., & Hirsch, P. (1999). Roseovarius tolerans gen. nov., sp. nov., a budding bacterium with variable bacteriochlorophyll a production from hypersaline Ekho Lake. International Journal of Systematic Bacteriology, 49, 137-147.

Lewin, R.A., Krienitz, L., Goericke, R., Takeda, H., & Hepperle, D. (2000). Picocystis salinarum gen. et sp. nov. (Chlorophyta) – a new picoplanktonic green alga. *Phycologia, 39, 560-565.*

Li, H.C., & Ku, T.L. (1997). δ^13^C-δ^18^O covariance as a paleohydrological indicator for closed-basin lakes. Palaeogeography, Palaeoclimateology, Palaeoecology, 133, 69-80.

Lopez-Perez, M., Ghai, R., Leon, M.J., Rodriguez-Olmos, A., Copa-Patiño, J.L., Soliveri, J., Sanchez-Porro, C., Ventose, A., & Rodriguez-Valera, F. (2013) Genomes of “Spiribacter,” a streamlined, successful halophilic bacterium. BMC Genomics, 14, 787-799.

Matsumoto, G. I., & Nagashima, H. (1984). Occurrence of 3-hydroxy acids in microalgae and cyanobacteria and their geochemical significance. *Geochimica et Cosmochimica Acta,* *48,* *1683–1687.*

Milford, A.D., Achenbach, L.A., Jung, D.O., & Madigan, M.T. (2000). Rhodobaca bogoriensis gen. nov. and sp. nov., an alkaliphilic purple nonsulfur bacterium from African Rift Valley soda lakes. Archives of Microbiology, 174, 18-27.

Oremland, R.S., Stolz, J.F., Hollibaugh, J.T. (2004). The microbial arsenic cycle in Mono Lake, California. FEMS Microbial Ecology, 48, 15-27.

Popp, B.N., Laws, E.A., Bidigare, R.R., Dore, J.E., Hanson, K.L., & Wakeham, S.G. (1998). Effect of phytoplankton cell geometry on carbon isotopic fractionation. Geochimica et Cosmochimica Acta, 62, 69-77.

Rodrigues, J.L.M., & Isanapong, J. (2014). The Family Opitutaceae. The Prokaryotes, 4, 751-756.

Roger, F., Bertilsson, S., Langenheder, S., Ahmed Osman, O. & Gamfeldt, L. (2016). Effects of multiple dimensions of bacterial diversity on functioning, stability and multifunctionality. *Ecology, 97, 2716-2728.*

Rua, C.P.J., Thompson, F. (2014). The Unclassified Genera of Gammaproteobacteria: Alkalimonas, Arenicella, Chromatocurvus, Congregibacter, Gallaecimonas, Halioglobus, Marinicella, Methylohalomonas, Methylonatrum, Orbus, Plasticicumulans, Porticoccus, Sedimenticola, Solimonas. The Prokaryotes, 3, 749-768.

Sobieraj, M., & Boone, D.R. (2006). Syntrophomonadaceae. The Prokaryotes, 3, 1041-1049.

Sorokin, D.Y., Abbas, B., Tourova, T.P., Bumazhkin, B.K., Kolganova, T.V., & Muyzer, G. (2014). Sulfate-dependent acetate oxidation under extremely natron-alkaline conditions by syntrophic associations from hypersaline soda lakes. Microbiology, 160, 723-732.

Sorokin, D.Y., Gorlenko, V.M., Tourova, T.P., Tsapin, A.I., Nealson, K.H., & Kuenen, G.J. (2002). Thioalkalimicrobium cyclicum sp. nov. and Thioalkalivibrio jannaschii sp. nov., novel species of haloalkaliphilic, obligately chemolithoautotrophic sulfur-oxidizing bacteria from hypersaline alkaline Mono Lake (California). International Journal of Systematic and Evolutionary Microbiology, 52, 913-920.

Sorokin, D.Y., Van Pelt, S., Tourova, T.P., & Evtushenko, L.I. (2009). Nitriliruptor alkaliphilus gen. nov., sp. nov., a deep-lineage haloalkaliphilic actinobacterium from soda lakes capable of growth on aliphatic nitriles, and proposal of Nitriliruptoraceae fam. nov. and Nitriliruptorales ord. nov. International Journal of Systematic and Evolutionary Microbiology, 59, 248-253.

Wilkes, E.B., & Pearson, A. (2019). A general model for carbon isotopes in red-lineage phytoplankton: Interplay between unidirectional processes and fractionation by RubisCO. Geochimica et Cosmochimica Acta, 265, 163-181.

Zeebe, R.E., & Wolf-Gladrow, D. (2001). CO2 in Seawater: Equilibrium, kinetics, isotopes (1^st^ ed).

Zhilina, T.N., Zavarzina, D.G., Kolganova, T.V., Tourova, T.P., & Zavarzin, G.A. (2005). "Candidatus contubernalis alkalaceticum," an obligately syntrophic alkaliphilic bacterium capable of anaerobic acetate oxidation in a coculture with Desulfonatronum cooperativum. Mikrobiologiia, 6, 800-809.
